# A multicomponent reaction-initiated synthesis of imidazopyridine-fused isoquinolinones

**DOI:** 10.3762/bjoc.21.92

**Published:** 2025-06-13

**Authors:** Ashutosh Nath, John Mark Awad, Wei Zhang

**Affiliations:** 1 Department of Chemistry, University of Massachusetts Boston, 100 Morrissey Boulevard, Boston, MA 02125, USA

**Keywords:** Groebke–Blackburn–Bienaymé (GBB), imidazopyridine, intramolecular Diels–Alder (IMDA), isoquinolinone, multicomponent reaction (MCR), re-aromatization

## Abstract

A new synthetic route initiated with Groebke–Blackburn–Bienaymé (GBB) followed by *N*-acylation, intramolecular Diels–Alder (IMDA), and dehydrative re-aromatization reactions for the synthesis of imidazopyridine-fused isoquinolinones is developed. Gaussian computation analysis on the effect of the substitution groups for the IMDA reaction is performed to understand the reaction mechanism.

## Introduction

Multicomponent reactions (MCRs) have intrinsic green chemistry advantages of synthetic efficiency and operational simplicity. Performing post-condensational modifications of MCRs could generate novel and complex molecular scaffolds [1–8]. Some MCR adducts generated from Ugi, Passerini, Gewald, Biginelli, and Groebke–Blackburn–Bienaymé (GBB) reactions have been modified to form chemically diverse heterocyclic scaffolds with potential biological activities [9–10].

**Figure 1:**
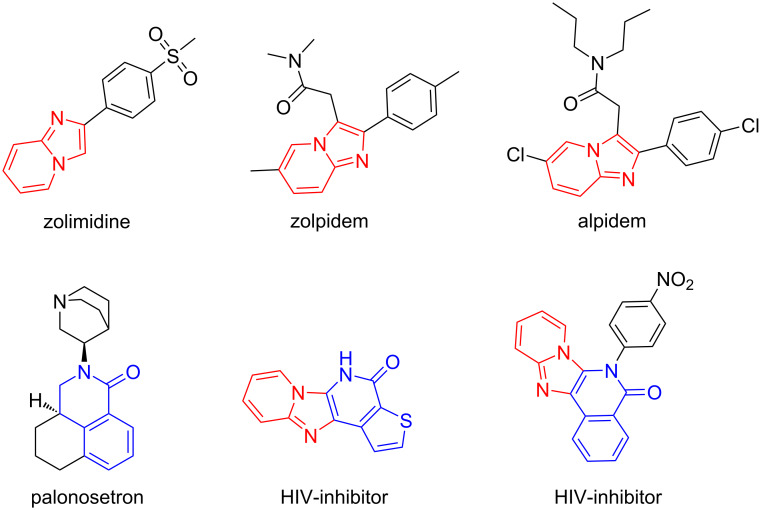
Bioactive compounds bearing imidazopyridine (red) and isoquinolinone-kind (blue) rings.

Imidazo[1,2-*a*]pyridine and isoquinolinone-kind scaffolds are privileged rings which can be found in drug molecules such as zolimidine [11], zolpidem [12], alpidem and antiemetic drug 5-HT3A antagonist palonosetron [13] (Figure 1). Imidazopyridine-fused isoquinolinones have been developed as HIV inhibitors [14]. The imidazo[1,2-*a*]pyridine ring can be readily synthesized by the GBB reaction [10,15], while the isoquinolinone ring is commonly generated by a cyclative lactamization process. Performing a GBB reaction followed by an intramolecular amidation is a good approach for making imidazopyridine-fused isoquinolinones.

**Scheme 1:**
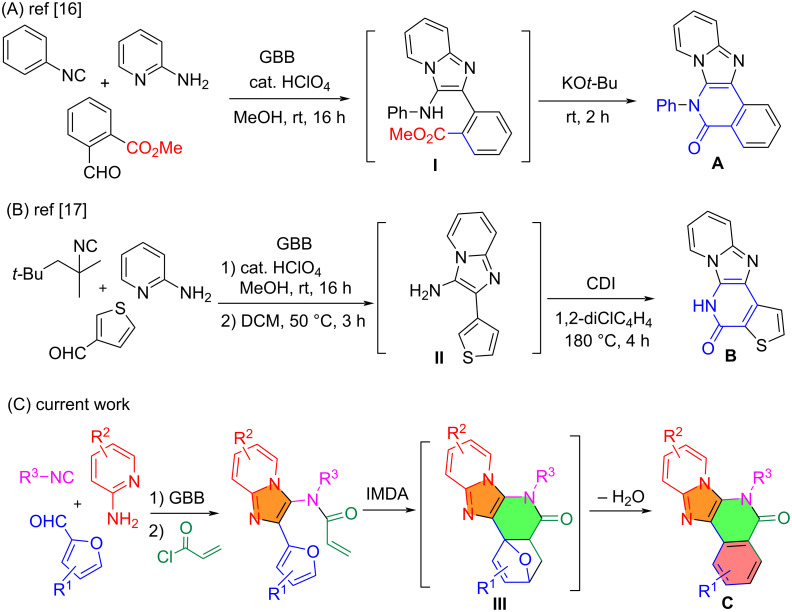
GBB-initiated synthesis of imidazopyridine-fused isoquinolinones.

The Veljkovic group employed methyl 2-formylbenzoate for the GBB reaction to form adducts **I** which undergoes intramolecular amidation to afford product **A** (Scheme 1A) [16]. In a patent filed by Tibotec Pharmaceuticals, substituted alkyl isonitriles were used for the GBB reaction followed by the cleavage of the alkyl group to give intermediate **II** as a free amine. Annulation of **II** with CDI gave product **B** which is an HIV reverse transcriptase inhibitor (Scheme 1B) [17]. We have reported a three-component [3 + 2] cycloaddition followed by IMDA reaction for making heterocyclic compounds [18]. Presented in this paper is a new synthetic route involving GBB, *N*-acylation and IMDA reactions for making intermediate **III** followed by dehydrative re-aromatization to give imidazopyridine-fused isoquinolinones **C** (Scheme 1C).

## Results and Discussion

**Scheme 2:**
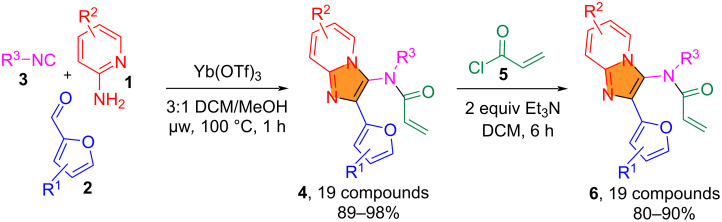
GBB reaction and *N*-acylation for the preparation of imidazo[1,2-*a*]pyridines **6**.

Following the reported procedures [10], the initial GBB reaction of aminopyridines **1** (0.5 mmol), isocyanides **3** (1.2 equiv), and furfuraldehydes **2** (1.2 equiv) was conducted in 3:1 CH_2_Cl_2_/MeOH (4 mL) using Yb(OTf)_3_ (0.08 equiv) as a Lewis acid catalyst under microwave irradiation at 100 °C for 1 h (Scheme 2). Nineteen distinct adducts **4** were obtained in 89–98% yields. Reactions of **4** with acryloyl chloride (**5**, 1.5 equiv) in the presence of Et_3_N (2 equiv) at room temperature in anhydrous CH_2_Cl_2_ for 6 h afforded 19 *N*-acylated compounds **6** in 80–90% yields [19].

With *N*-acylated GBB adducts **6** in hand, the synthesis of imidazopyridine-fused isoquinolinones **8** was explored by conducting IMDA and spontaneous dehydrative re-aromatization reactions. The IMDA reaction using **6a** as a model compound was systematically evaluated by varying catalysts, solvents, reaction temperatures and times (Table 1). The best conditions were found to use AlCl_3_ as a catalyst in 1,2-dichlorobenzene at 180 °C for 4 h, which gave **8a** in 85% conversion and 82% isolated yield (Table 1, entry 3). Other solvents like toluene and xylene gave minimal or no product. Different combinations of temperature and reaction time couldn’t improve the yield. Among the various Lewis acids tested, AlCl₃ gave the best result, while CuCl, ZnCl_2_, PdCl_2_ and Sc(OTf)_3_ showed moderate conversions (30–55%), and InCl_3_ had the lowest efficiency. Without any Lewis acid we observed no conversion by LC–MS (Table 1, entry 16). During the reaction, IMDA adduct **7a** was detected by LC–MS (Figure S1, Supporting Information File 1), but it was not stable enough for isolation. The structure of **8a** was confirmed by single crystal X-ray diffraction analysis.

**Table 1:** Optimization of IMDA and re-aromatization reactions for the preparation of **8a**.

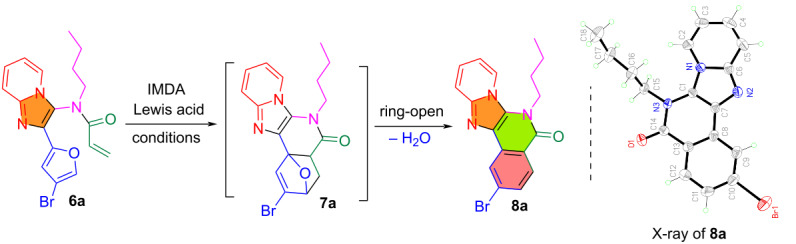					

entry	catalyst (10 mol %)	solvent	temp (°C)	time	conversion (%)

1	AlCl_3_	toluene	120	12 h	0
2	FeCl_3_	1,2-dichlorobenzene	120 (μw)	1 h	0
**3**	**AlCl** **_3_**	**1,2-dichlorobenzene**	**180**	**4 h**	**85**
4	AlCl_3_	1,2-dichlorobenzene	180 (μw)	1 h	5
5	AlCl_3_	1,2-dichlorobenzene	120 (μw)	2 h	15
6	AlCl_3_	xylene	140	4 h	0
7	ZnCl_2_	1,2-dichlorobenzene	140	4 h	50
8	CuCl	1,2-dichlorobenzene	180	4 h	55
9	PdCl_2_	1,2-dichlorobenzene	180	4 h	40
10	CsF	1,2-dichlorobenzene	180	4 h	30
11	Sc(OTf)_3_	1,2-dichlorobenzene	180	4 h	35
12	CsCO_3_	1,2-dichlorobenzene	180	4 h	30
13	InCl_3_	1,2-dichlorobenzene	180	4 h	20
14	Yb(OTf)_3_	1,2-dichlorobenzene	180	4 h	60
15	NiCl_2_	1,2-dichlorobenzene	180	4 h	47
16	no catalyst	1,2-dichlorobenzene	180	4 h	0

**Scheme 3:**
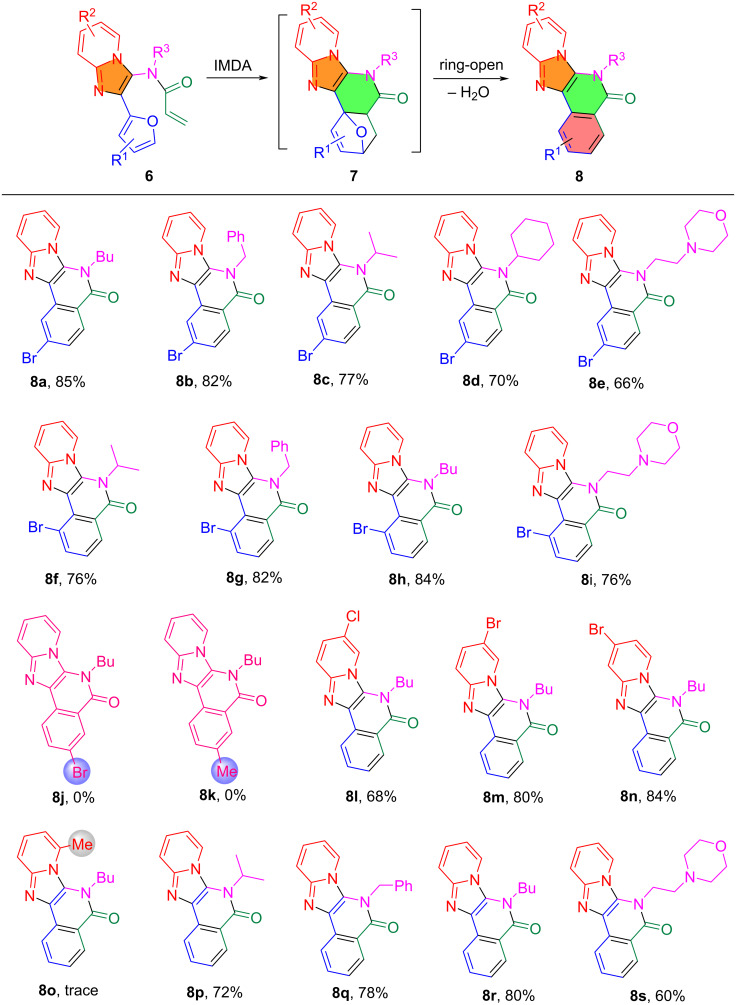
Substrate scope for IMDA and dehydrative aromatization in making **8**. Reaction conditions: **6** and AlCl_3_ (10 mol %) in 1,2-dichlorobenzene at 180 °C for 4 h.

**Figure 2:**
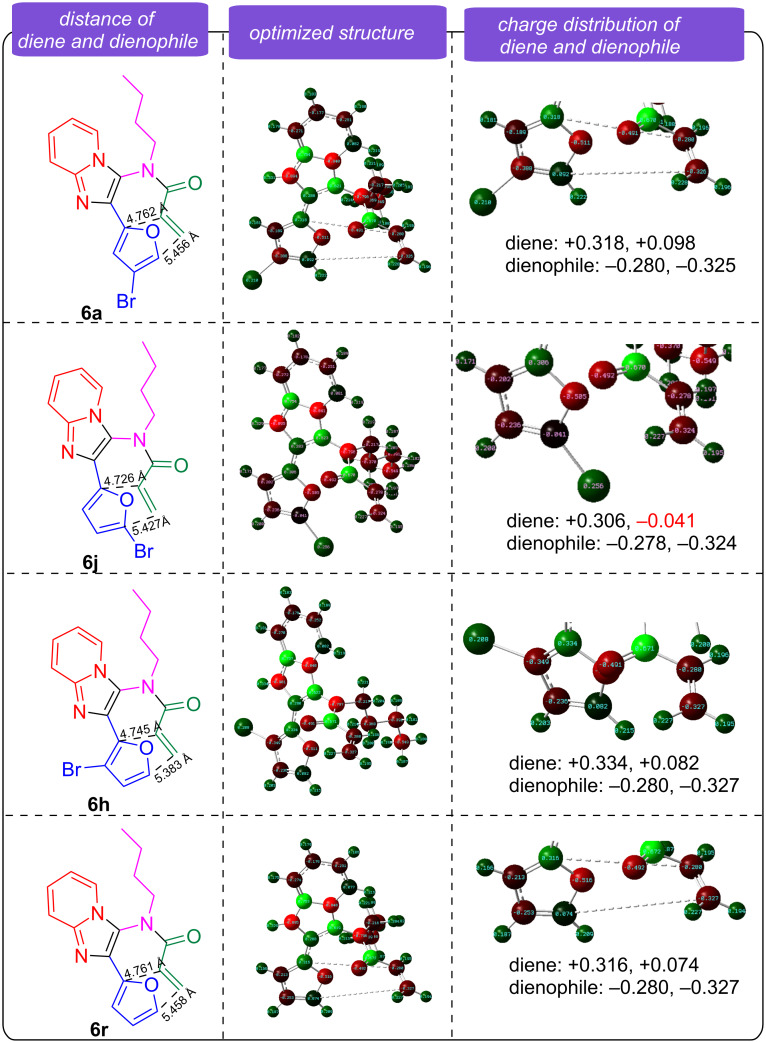
Transition state analysis of IMDA reactions for **6a**, **6j**, **6h** and **6r**.

The optimized reaction conditions were used to evaluate the substrate scope of the synthesis of imidazopyridine-fused isoquinolinones **8** (Scheme 3). The R^1^ residue on the furan ring was found to have the most significant impact on the IMDA reaction. A bromine atom at the 3- or the 4-position resulted in products **8a**–**i** in 66–86% yields, while a bromine atom or a methyl group at the 5-position inhibited the IMDA reaction in the preparation of **8j** and **8k**. A comprehensive DFT investigation of reactant **6** was carried out to analyze the transition state of the IMDA reaction for a Br-substituted diene and its charge distribution (Figure 2). The diene has a notable positive charge (+0.318, +0.098, **6a**), (+0.334, +0.082, **6h**) and (+0.316, +0.074, **6r**) whereas the dienophile presents a negative charge (−0.280 to −0.325, **6a**), (−0.280 to −0.327, **6h**) and (−0.280 to −0.327, **6r**), respectively. This structure induces electrostatic repulsion instead of the requisite attraction for a successful interaction between the electron-rich diene and the electron-deficient dienophile, characteristic of Diels–Alder processes. The incorporation of a bromine atom at the 5-position of the diene (+0.306, −0.041, **6j**) complicates the situation. As an electronegative element, Br exerts an inductive electron-withdrawing influence to enhance the electron shortage of the diene. This electronic imbalance reduces the diene's nucleophilicity, rendering it less reactive to the dienophile. The unfeasibility of the IMDA reaction in this system arises from inadequate interatomic distances, electrostatic repulsion from incompatible associated dienophile was conducted [19–20]. Firstly, the charge and the electronic consequences of the 5-Br substitution **6j** were considered, which were found to inhibit the system from attaining the requisite conditions for successful cycloaddition. Secondly, the interatomic distances between the reactive centers of the diene and dienophile are almost similar for all substitutes of **6a**, **6h**, **6r** and **6j**, which, are not ideal effective for IMDA cycloadditions compared to the other substitute cycloadditions.

The R^2^ substituent on the imidazopyridine moiety in **6** was found to have a significant electronic impact on the IMDA cycloaddition. When R^2^ is a halogen (Br or Cl), it withdraws electron density through its inductive (−I) effect to increase diene reactivity for the cycloaddition to form **7**. For example, **6l** (R^2^ = 6-Cl, 68% yield of **8l**), **6m** (R^2^ = 6-Br, 80% yield of **8m**), and **6n** (R^2^ = 7-Br, 84% yield of **8n**) are high-yielding substrates. But an electron-donating group in **6o** (R^2^ = 5-methyl) lowers the dienophilic nature and gave no product **8o**. The R^3^ substituent from isocyanides is an important factor in forming intermediates **7** and promoting dehydrative aromatization for making products **8**. The reactions with R^3^ = *n*-butyl resulted in the high yielding formation of **8a**,**h**,**l**,**m**,**n** and **8r** (68–85%), R^3^ = phenyl resulted in **8b**,**g** and **8q** in 78–82% yields, R^3^ = isopropyl and cyclopentene gave **8c**,**f**,**p** and **8d** in greater than 70% yields, and R^3^ = 2*-*morpholinoethyl gave **8e**,**i** and **8s** in 60–76% yields.

**Figure 3:**
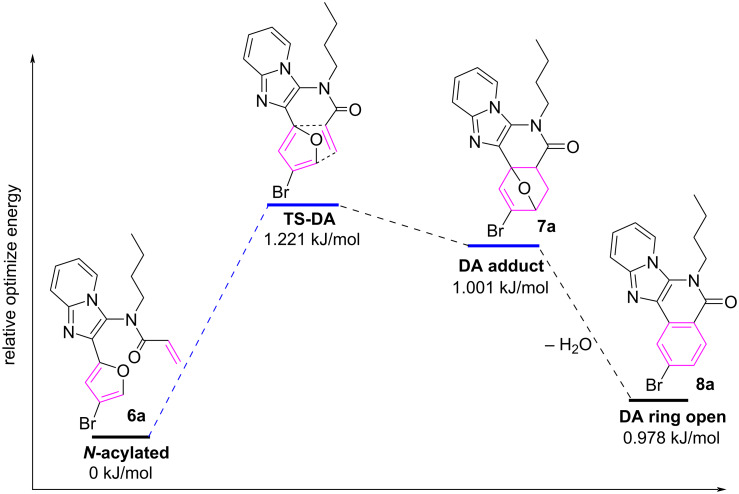
Relative energy diagram for the synthesis of **8a** from **6a**.

The energy status for the transformation of compound **6a** to **8a** was calculated using the Gaussian 16 software (Figure 3) [21]. The *N*-acylated compound **6a** has a baseline relative energy of 0 kJ/mol, while the transition state of the Diels–Alder (**TS-DA**) reaction presents the highest energy barrier at 1.221 kJ/mol. The DA adduct shows a little lower energy at 1.001 kJ/mol, indicating a smooth transition from the transition state to the product. The final dehydrative ring-opening gives products by decreasing the energy to 0.978 kJ/mol. Computational analysis indicates that the IMDA step has a high energy barrier which needs a catalyst, while the dehydrative re-aromatization step is energetically favorable.

**Scheme 4:**
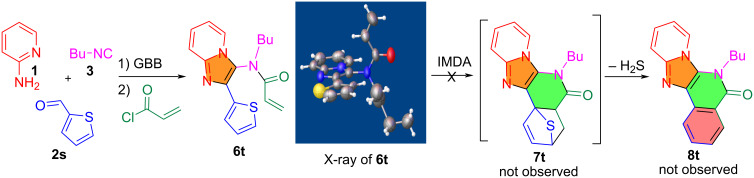
Using thiophene-2-carbaldehyde for the synthesis of **8t**.

Other than furfural, thiophene-2-carbaldehyde (**2s**) was used for the GBB and *N*-acylation reactions to make **6t** (Scheme 4). The IMDA reaction of **6t** was carried out under the catalysis of AlCl_3_ in dichlorobenzene at 180 °C for up to 24 h, but no compounds **7t** and **8t** could be detected by LC–MS from the reaction mixture. The X-ray structure of **6t** indicated that the diene and dienophile are perpendicular to each other which prevents them from being properly aligned for the IMDA reaction. The transition state of the IMDA is electronically destabilized by the sulfur group of the thiophene to reduce the diene's reactivity or altering the electrophilicity of the dienophile.

**Scheme 5:**
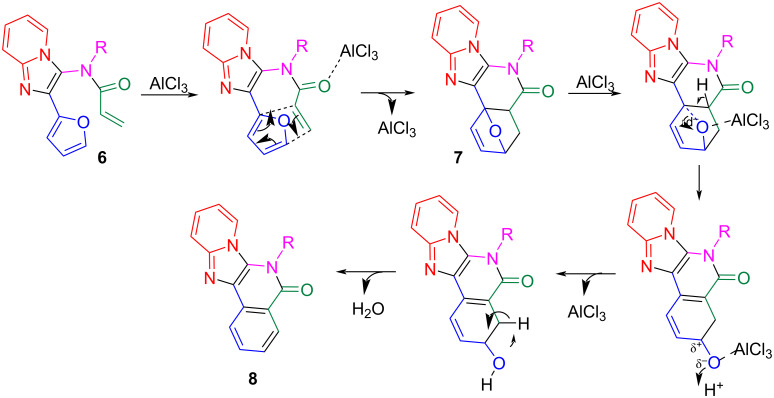
Proposed mechanisms for IMDA reaction and dehydration re-aromatization.

Based on the computational analysis of the transition states, reaction mechanisms for the IMDA and the dehydration re-aromatization process are proposed in Scheme 5. In the IMDA reaction for the preparation of intermediate **7**, the carbonyl oxygen interacts with AlCl₃, enhancing the electrophilicity and promoting the rearrangement to form stable oxonium ions. The removal of water from **7** is facilitated by protonation, producing reactive carbocations which undergo dehydrative aromatization to produce products **8**.

## Conclusion

In summary, we developed a reaction sequence involving GBB, *N*-acylation, IMDA and dehydrative re-aromatization reactions for the synthesis of imidazopyridine-fused isoquinolinones. Computational studies of the IMDA reaction indicated that the position of the R^1^ group on the furan ring and the R^2^ group on the imidazopyridine moiety have direct electronic impact on the IMDA reaction. This integrated reaction process provided a new avenue for the preparation of heterocyclic scaffolds with potential biological activity.

## Experimental

### General procedure for the synthesis of intermediates **4** and **6**

The GBB reactions for the preparation of imidazo[1,2-*a*]pyridines **4** were conducted using aminopyridines **1** (0.5 mmol), isocyanides **3** (0.6 mmol, 1.2 equiv), and furfuraldehyde **2** (0.6 mmol, 1.2 equiv) in 3:1 DCM/MeOH (4 mL) with Yb(OTf)_3_ (0.04 mmol, 0.08 equiv) as a Lewis acid catalyst under microwave irradiation at 100 °C for 1 h (Scheme 2, Table S1 in Supporting Information File 1). Nineteen distinct adducts **4** were obtained in 89–98% yields. The reactions of GBB adducts **4** with acryloyl chloride (**5**, 1.5 equiv) in the presence of Et_3_N (2 equiv) at room temperature in anhydrous CH_2_Cl_2_ for 6 h afforded 19 *N*-acylated compounds **6** in 80–90% yields after flash chromatography with 1:6 EtOAc/hexanes (Scheme 2, Table S2 in Supporting Information File 1) [19].

### General procedure for the synthesis of products **8**

In the presence of 0.08 equiv of Lewis’s acid AlCl_3, _*N*-acylation products **6** (0.1 mmol) in dichlorobenzene were heated at 180 °C for 4 h (Scheme 3). The reaction mixtures were checked by LC–MS to follow the formation of DA adducts **7** and the ring opening products **8** (Figure S1, Supporting Information File 1). After 4 h, the reaction mixtures were worked up and the crude products were purified by flash chromatography with 30:70 EtOAc/hexanes. Product structures were confirmed by ^1^H and ^13^C NMR analysis and X-ray crystal structure analysis of **8a**.

### Density functional theory (DFT) calculations

DFT computations were conducted utilizing Gaussian 16W with the B3LYP functional and the 6-31G(d,p) basis set [21–22]. Geometry optimizations were performed without symmetry restrictions, and frequency analyses verified that all structures represented genuine minima. Charge distributions and interatomic distances were evaluated to determine reaction feasibility, utilizing GaussView for molecular visualization.

## Supporting Information

File 1 General reaction procedures, compound characterization data, and copies of NMR spectra.

## Data Availability

All data that supports the findings of this study is available in the published article and/or the supporting information of this article.
