# Supplementary material for: A multicomponent reaction-initiated synthesis of imidazopyridine-fused isoquinolinones
Source: Beilstein J Org Chem. 2025 Jun 13;21:1161–9. doi: 10.3762/bjoc.21.92 (PMC12207252; doi:10.3762/bjoc.21.92)
Supplement: File 1 — General reaction procedures, compound characterization data, and copies of NMR spectra. [file Beilstein_J_Org_Chem-21-1161-s001.pdf]

BEILSTEIN JOURNAL OF ORGANIC CHEMISTRY

Supporting Information

for

A multicomponent reaction-initiated synthesis of imidazopyridine-fused isoquinolinones

Ashutosh Nath, John Mark Awad and Wei Zhang

Beilstein J. Org. Chem. **2025,** *21,* 1161–1169. doi:10.3762/bjoc.21.92

General reaction procedures, compound characterization data, and copies of NMR spectra

License and Terms: This is a supporting information file under the terms of the Creative Commons Attribution License (https://creativecommons.org/licenses/by/4.0). Please note that the reuse, redistribution and reproduction in particular requires that the author(s) and source are credited and that individual graphics may be subject to special legal provisions.
The license is subject to the *Beilstein Journal of Organic Chemistry* terms and conditions: (https://www.beilstein-journals.org/bjoc/terms)

Table of Contents

General Information…………………………………………………………..………....S2

General procedure for the synthesis of GBB products **4** ……………………….…...….S2

General procedure of N-acylation for the synthesis of products **6** ………………...……S2

General procedure for IMDA and dehydrative re-aromatization products **8** ………...….S2

X-ray crystelography report of **8a** and **6t** …………………………..………….…..…..S7

Analytical characterization data of products **6a** and **8** …………………………………..S9

^{1}H NMR, ^{13}C NMR spectra of products **6a** and 8 ………………………………….…..S13

General Information.

All first reactions were conducted in a sealed Biotage microwave reaction vial containing 2–5 mL, unless stated otherwise. Analytical thin layer chromatography (TLC) was conducted on silica gel plates including an F-254 indicator, and chemicals were seen using UV light irradiation. Column chromatography was performed utilizing silica gel (200−300 mesh) under elevated pressure. The ^{1}H, and ^{13}C spectroscopic data were acquired using Bruker Mercury Plus 400 MHz or Bruker AVANCE NEO 500 MHz NMR spectrometers. Chemical shifts were expressed in parts per million (ppm) relative to internal TMS for 1H NMR data and deuterated solvent for ^{13}C NMR data. ^{1}H NMR coupling constants were expressed in Hz, with multiplicity denoted as follows: s (singlet); d (doublet); t (triplet); q (quartet); m (multiplet); dd (doublet of doublets); and td (triplet of doublets). LC–MS were performed on an Agilent 2100 system with C_{18} column (5.0 μm, 6.0 × 50 mm). The mobile phases were ACN and H_2O both containing 0.05% trifluoroacetic acid. A linear gradient was used to increase from 25:75 (v/v) $MeOH/H_2O$ to 100% MeOH in 7.0 min at a flow rate of 0.7 mL/min. UV detections were conducted at 210 nm, 254 nm and 280 nm.

General procedure for the synthesis of GBB product 4

The initial GBB reactions for making imidazo[1,2-*a*] pyridines **4** were conducted using aminopyridines **1** (0.5 mmol), isocyanides **3** (0.6 mmol, 1.2 equiv), and furfuraldehyde **2** (0.6 mmol, 1.2 equiv) in 3:1 DCM/MeOH (4 mL) were conducted using $Yb(OTf)_3$ (0.04 mmol, 0.08 equiv) as a Lewis acid catalyst under microwave irradiation at 100 °C for 1 h (Scheme 2, Table S1). Nineteen distinct adducts **4** were obtained in 89–98% yields.

General procedure of *N*-acylation for the synthesis of product 6

Reactions of **4** with acryloyl chloride **5** (1.5 equiv) in the presence of Et_3N (2 equiv) at room temperature in anhydrous CH_2Cl_2 for 6 h afforded 19 *N*-acylated compounds **6** in 80–90% yields (Table S2) [10]. Further purification was conducted by flash chromatography with 1:6 EtOAc/*n*-hexane. The adduct was confirmed by NMR.

General procedure for IMDA and dehydrative re-aromatization product 8

After step-2 isolated *N*-acylation product **6** and reflux in dichlorobenzene solvent 4 h at 180 °C with 0.08 equiv. Lewis's acid $AlCl_3$ (Table S3). During this reaction we checked crude LC–MS and observed DA-adduct **7** and ring open product **8**. After 4 h reaction isolated ring open product **8** and purified with Ethyl acetate/hexane (30:70). We confirmed product structure from ^{1}H, ^{13}C NMR and x-ray crystal structure analysis.

Table S1: Three-component GBB cycloaddition for the syntheses of **4**.

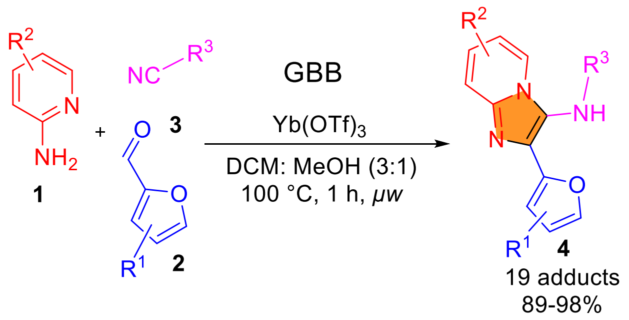

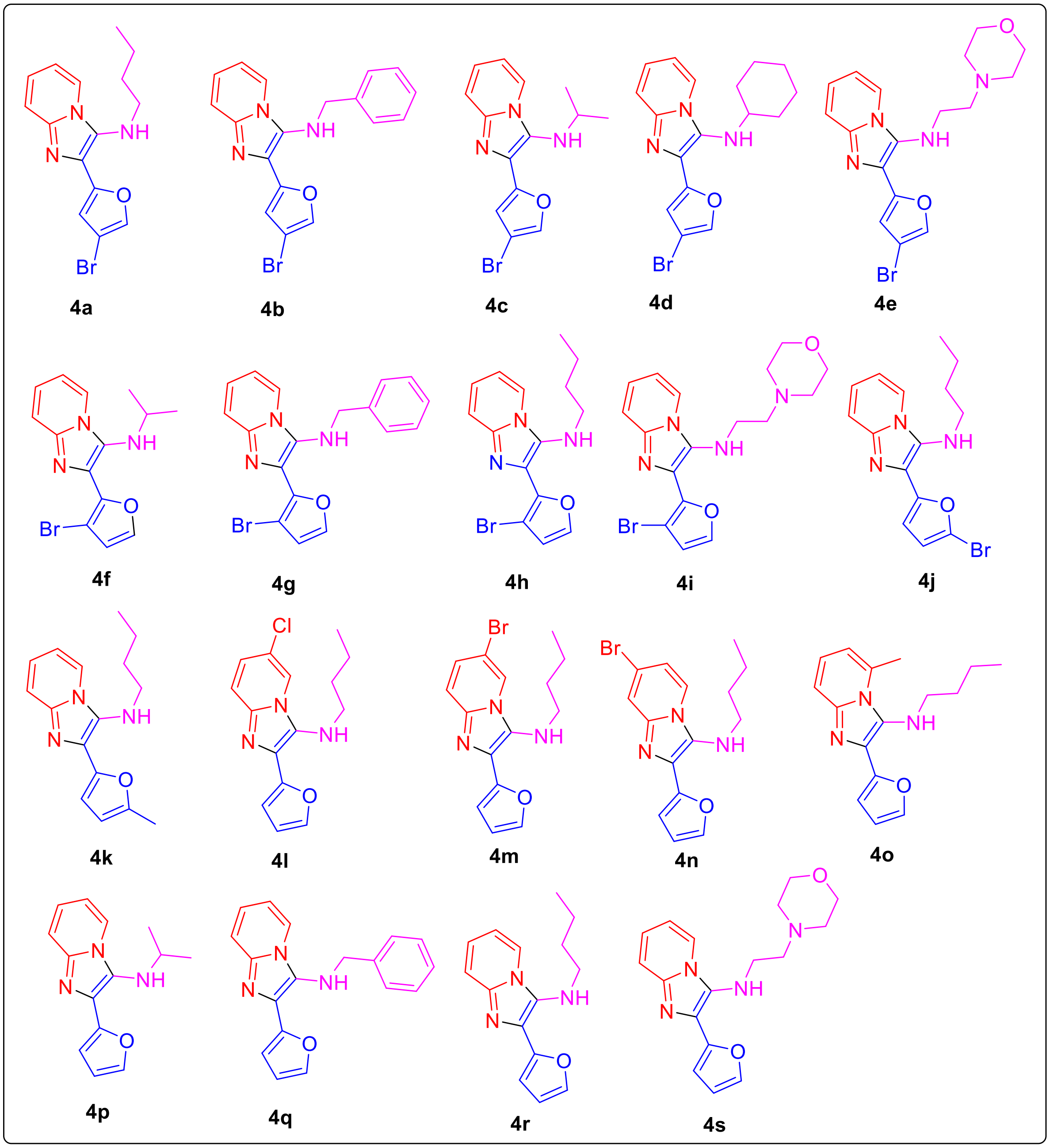

Table S2: *N*-Acylation of fused imidazo[1,2-*a*]pyridines.

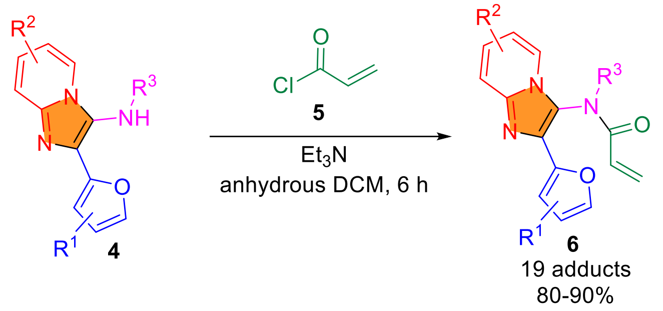

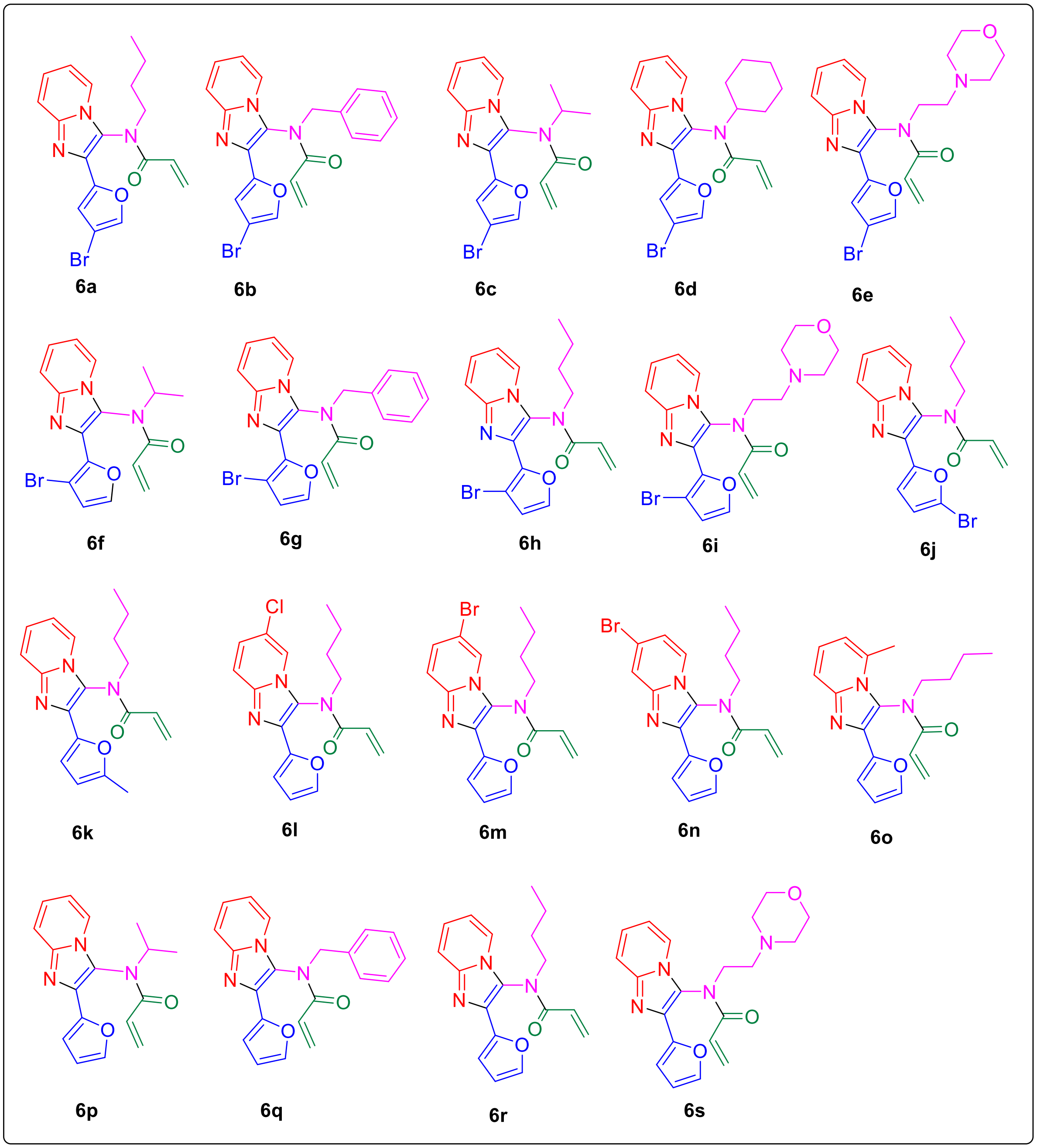

Table S3: Substrate scope for the reaction of imidazopyridine-fused isoquinolinones **8**.

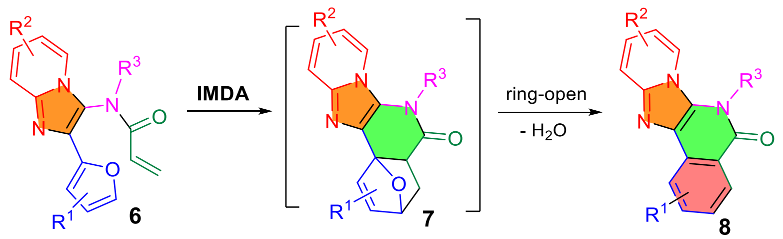

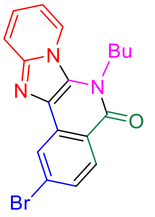

8a, 85%

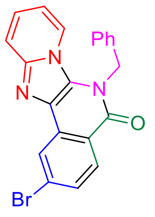

8b, 82%

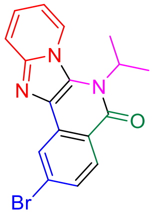

8c, 77%

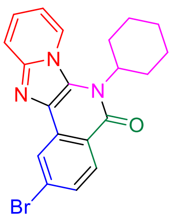

8d, 70%

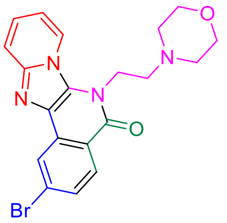

8e, 66%

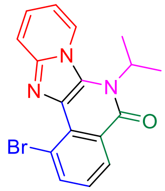

8f, 76%

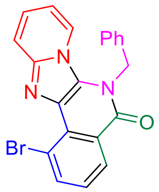

8g, 82%

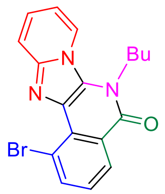

8h, 84%

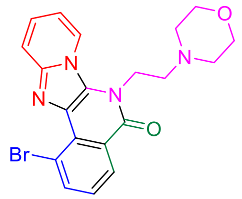

8i, 76%

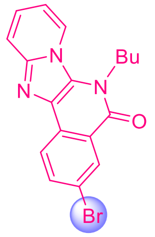

8j, 0%

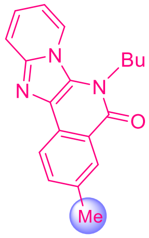

8k, 0%

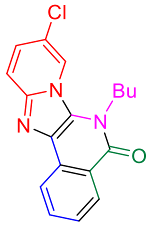

8l, 68%

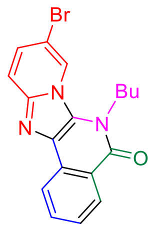

8m, 80%

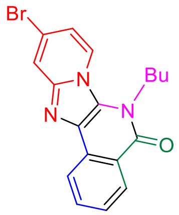

8n, 84%

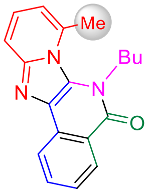

8o, trace

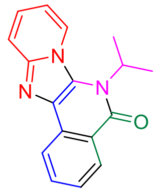

8p, 72%

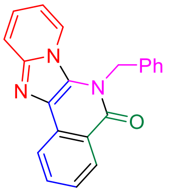

8q, 78%

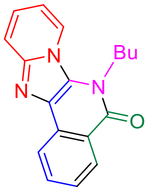

8r, 80%

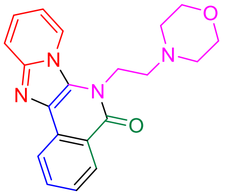

8s, 60%

[a] Reactions of **6** were carried out using $AlCl_3$ (10 mol %) in 1,2-dichlorobenzene at 180 °C for 4 h.

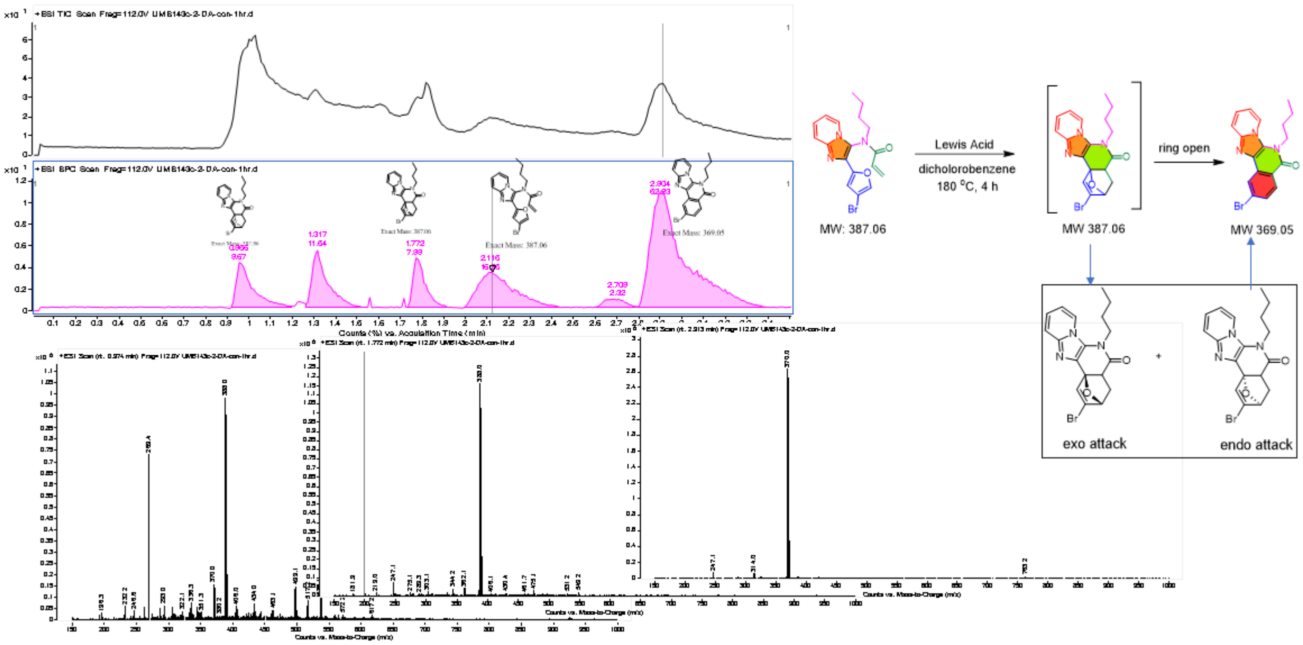

Figure S1: LC–MS of overserving IMDA Adduct **7a**.

X-ray crystallographic analysis of 8a (CCDC: 2429172)

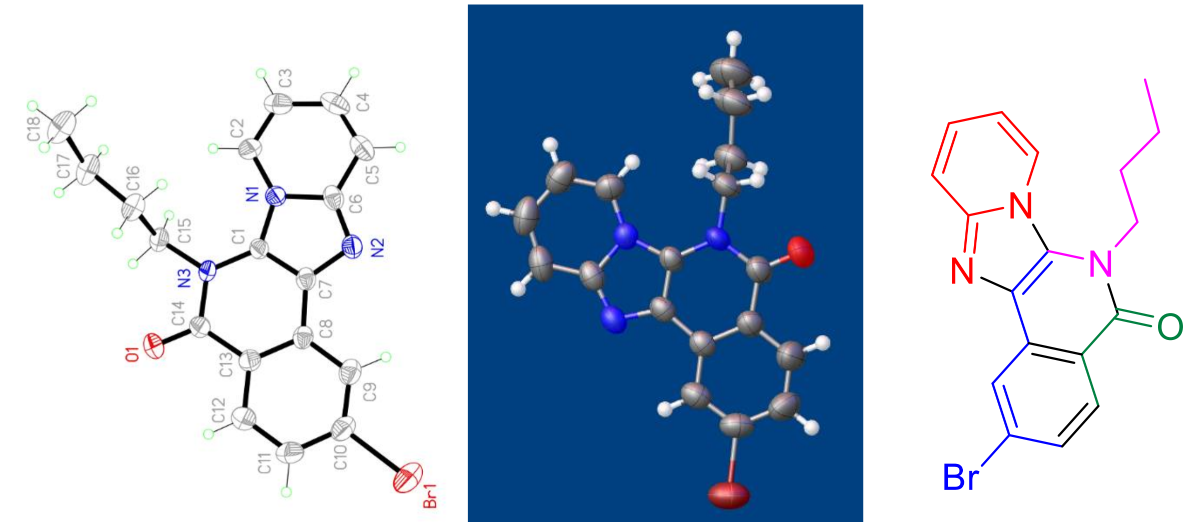

8a

Cell	a = 19.882(3) Å; b = 7.8944(9) Å; c = 20.396(3) Å<br>a= 90°; b= 90°; g = 90°.	
Temperature	300(2) K	
	Calculated	Reported
Volume	3201.3(7) $Å^3$	3201.3(7) $Å^3$
Space group	Pbca	Pbca
Moiety formula	$C_{18}H_{16}BrN_3O$	$C_{18}H_{16}BrN_3O$
Sum formula	$C_{18}H_{16}BrN_3O$	$C_{18}H_{16}BrN_3O$
Z	8	8
μ (mm^{-1})	2.576	2.576
F000	1504	1504
**CCDC: 2429172**		

X-ray crystallographic analysis of 6t (CCDC: 2429579)

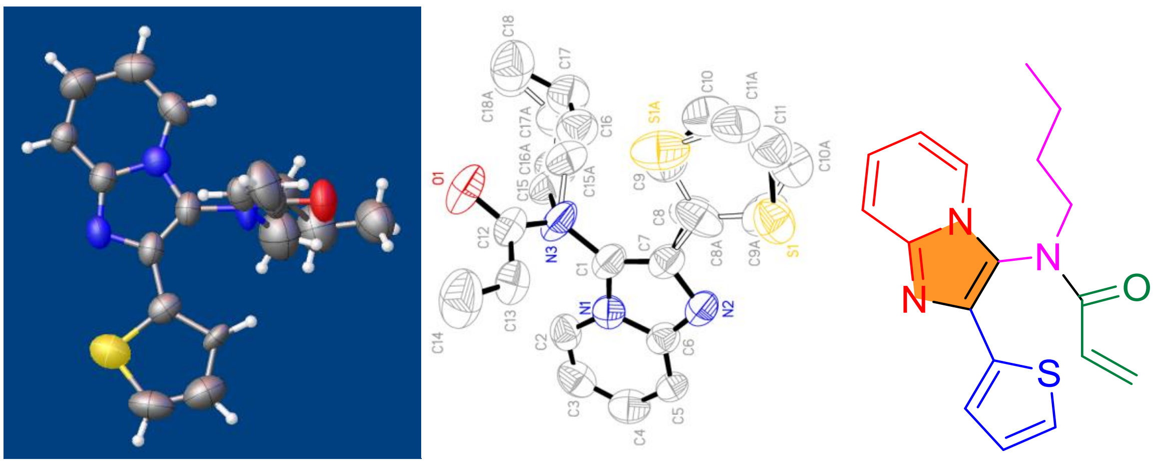

6t

Cell	a = 11.5629(12) Å; b = 11.0333(12) Å; c = 14.0321(16) Å<br>a= 90°; b= 106.650°; g = 90°.	
Temperature	300(2) K	
	Calculated	Reported
Volume	1715.1(3) $Å^3$	1715.1(3) $Å^3$
Space group	$P2_1/n$	$P2_1/n$
Moiety formula	$C_{18}H_{19}N_3OS$	$C_{18}H_{19}N_3OS$
Sum formula	$C_{18}H_{19}N_3OS$	$C_{18}H_{19}N_3OS$
Z	4	4
μ (mm^{-1})	2.576	2.576
F000	1504	1504
**CCDC: 2429579**		

Analytical characterization data of products **6a** and **8**.

N-(2-(4-Bromofuran-2-yl)imidazo[1,2-a]pyridin-3-yl)-N-butylacrylamide (**6a**)

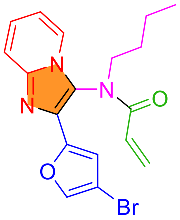
6a

Light yello solid (90% yield), Chemical Formula: $C_{18}H_{18}BrN_3O_2$ ^{1}H NMR (400 MHz, cdcl$_3$) δ 7.79 (d, J = 6.8 Hz, 1H), 7.63 (d, J = 9.1 Hz, 1H), 7.48 (s, 1H), 7.36 – 7.26 (m, 1H), 6.92 (t, J = 6.8 Hz, 1H), 6.81 (s, 1H), 6.43 (d, J = 16.7 Hz, 1H), 5.80 (dd, J = 16.7, 10.3 Hz, 1H), 5.50 (d, J = 10.4 Hz, 1H), 3.87 (ddd, J = 15.5, 10.1, 5.7 Hz, 1H), 3.67 (td, J = 13.3, 11.9, 5.9 Hz, 1H), 1.45 – 1.20 (m, 5H), 0.97 – 0.81 (m, 3H).

2-Bromo-6-butylpyrido[2',1':2,3]imidazo[4,5-c]isoquinolin-5(6H)-one (**8a**)

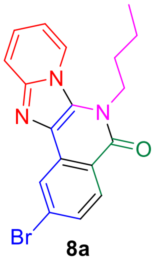
8a

Yellow solid (85% yield), Chemical Formula: $C_{18}H_{16}BrN_3O$. ^{1}H NMR (400 MHz, cdcl$_3$) δ 8.57 (d, J = 1.9 Hz, 1H), 8.37 – 8.30 (m, 2H), 7.69 (d, J = 9.2 Hz, 1H), 7.63 (dd, J = 8.7, 1.9 Hz, 1H), 7.26 – 7.15 (m, 2H), 6.87 (t, J = 7.0 Hz, 1H), 4.63 (t, J = 7.9 Hz, 2H), 1.88 (q, J = 7.9 Hz, 2H), 1.59 – 1.50 (m, 2H), 1.02 (t, J = 7.4 Hz, 3H). ^{13}C NMR (100 MHz, cdcl$_3$) δ 158.5, 136.25, 134.2, 127.2, 124.1, 123.0, 122.6, 120.1, 118.1, 118.0, 116.2, 114.4, 112.0, 107.2, 41.2, 29.9, 17.9, 11.7. HRMS (ESI) calcd for $C_{18}H_{16}BrN_3O$ m/z): 369.0477, found 369.0480.

6-Benzyl-2-bromopyrido[2',1':2,3]imidazo[4,5-c]isoquinolin-5(6H)-one (**8b**)

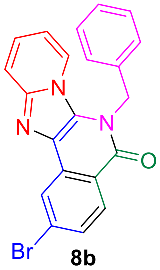
8b

Light yellow solid (82% yield), $C_{21}H_{14}BrN_3O$. ^{1}H NMR (399 MHz,) ^{1}H NMR (399 MHz, cdcl$_3$) δ 8.55 (d, J = 2.0 Hz, 1H), 8.40 – 8.31 (m, 5H), 7.70 – 7.61 (m, 4H), 7.17 (dd, J = 9.6, 1.8 Hz, 2H), 4.61 (t, J = 7.9 Hz, 2H). ^{13}C NMR (100 MHz, cdcl$_3$) δ 150.1, 137.2, 130.3, 130.2, 128.9, 128.8, 126.3, 126.3, 125.1, 121. 8, 120.8, 119.9, 105.1, 104.9, 46.6. HRMS (ESI) calcd for $C_{21}H_{14}BrN_3O$ m/z): 403.0320, found 403.0341.

2-Bromo-6-isopropylpyrido[2',1':2,3]imidazo[4,5-c]isoquinolin-5(6H)-one (**8c**)

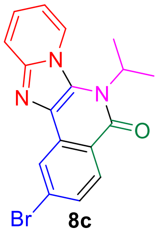
8c

Off-white solid (77% yield), Chemical Formula: $C_{17}H_{14}BrN_3O$. ^{1}H NMR (399 MHz,) δ 8.57 (d, J = 1.9 Hz, 1H), 8.37 – 8.30 (m, 2H), 7.69 (d, J = 9.2 Hz, 1H), 7.63 (dd, J = 8.7, 1.9 Hz, 1H), 7.26 – 7.15 (m, 1H), 6.87 (t, J = 7.0 Hz, 1H), 5.09 – 4.75 (m, 1H), 1.89 – 1.83 (m, 6H). ^{13}C NMR (100 MHz, cdcl$_3$) δ 168.3, 148.2, 131.9, 125.1, 124.1, 120.9, 120.1, 111.5, 110.2, 108.2, 106.3, 44.9, 20.1. HRMS (ESI) calcd for $C_{17}H_{14}BrN_3O$ m/z): 355.0320, found 355.0624.

2-Bromo-6-cyclohexylpyrido[2',1':2,3]imidazo[4,5-c]isoquinolin-5(6H)-one (**8d**)

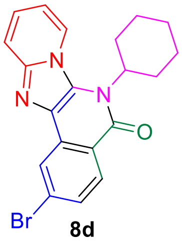
8d

Off-white solid (70% yield), Chemical Formula: $C_{20}H_{18}BrN_3O$. ^{1}H NMR (399 MHz, cdcl$_3$) δ 8.38 – 8.31 (m, 1H), 8.26 (d, J = 8.5 Hz, 0H), 8.14 (d, J = 7.4 Hz, 1H), 7.69 (d, J = 9.3 Hz, 1H), 7.50 – 7.43 (m, 1H), 7.18 (s, 0H), 6.89 (t, J = 7.0 Hz, 1H), 4.39 (d, J = 12.1 Hz, 1H), 2.95 – 2.85 (m, 2H), 1.99 (dd, J = 26.2, 11.4 Hz, 5H), 0.85 (d, J = 17.3 Hz, 3H). ^{13}C NMR (100 MHz, cdcl$_3$) δ 167.4, 143.1, 135.9, 135.2, 130.6, 128.0, 126.6, 126.3, 125.9, 124.9, 121.6, 117.9, 113.4, 48.2, 30.6, 20.3, 13.7.

2-Bromo-6-(2-morpholinoethyl)pyrido[2',1':2,3]imidazo[4,5-c]isoquinolin-5(6H)-one (**8e**)

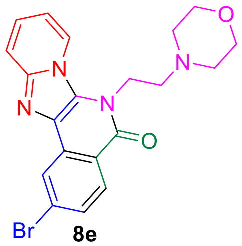
8e

Yellow solid (66% yield), Chemical Formula: $C_{20}H_{19}BrN_4O_2$, ^{1}H NMR (399 MHz, cdcl$_3$) δ 8.72 (d, *J* = 7.9 Hz, 1H), 8.01 (s, 1H), 7.69 (s, 1H), 7.25 (d, *J* = 2.0 Hz, 4H), 4.74 (d, *J* = 24.6 Hz, 2H), 3.64 (s, 2H), 3.34 (s, 4H), 2.95 (d, *J* = 1.9 Hz, 4H). ^{13}C NMR (100 MHz, cdcl$_3$) δ 167.2, 147.2, 143.5, 130.3, 126.7, 121.9, 120.1, 119.9, 117.4, 111.6, 109.2, 63.2, 45.38, 44.7, 35.2, 28.7.

1-Bromo-6-isopropylpyrido[2',1':2,3]imidazo[4,5-c]isoquinolin-5(6H)-one (**8f**)

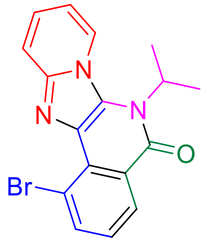
8f

Off-white solid (76% yield), Chemical Formula: $C_{17}H_{14}BrN_3O$. ^{1}H NMR (399 MHz,) δ 8.52 – 8.46 (m, 1H), 8.23 (d, *J* = 7.2 Hz, 1H), 8.03 (d, *J* = 8.4 Hz, 1H), 7.80 (d, *J* = 9.2 Hz, 1H), 7.36 (t, *J* = 7.9 Hz, 1H), 7.25 (d, *J* = 1.2 Hz, 1H), 6.88 (t, *J* = 7.0 Hz, 1H), 5.09 – 4.75 (m, 1H), 1.89 – 1.83 (m, 6H). ^{13}C NMR (100 MHz, cdcl$_3$) δ 143.1, 129.3, 128.1, 126.0, 122.1, 118.5, 116.0, 113.5, 49.6, 20.8, 19.8. HRMS (ESI) calcd for $C_{17}H_{14}BrN_3O$ m/z): 355.0320, found 355.0624.

6-Benzyl-1-bromopyrido[2',1':2,3]imidazo[4,5-c]isoquinolin-5(6H)-one (**8g**)

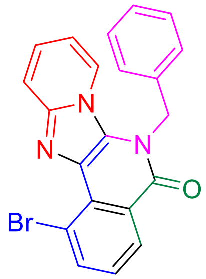
8g

Light yellow solid (82% yield), Chemical Formula: $C_{21}H_{14}BrN_3O$. ^{1}H NMR (400 MHz, cdcl$_3$) δ 7.53 (dt, *J* = 9.1, 1.2 Hz, 1H), 7.42 (dd, *J* = 3.7, 1.1 Hz, 1H), 7.36 (dd, *J* = 5.1, 1.1 Hz, 1H), 7.21 – 7.04 (m, 6H), 7.01 (dt, *J* = 6.8, 1.2 Hz, 1H), 6.55 – 6.44 (m, 2H), 4.61 (t, *J* = 7.9 Hz, 2H). ^{13}C NMR (100 MHz, cdcl$_3$) δ 139.5, 135.5, 129.5, 129.5, 128.0, 127.4, 125.4, 123.6, 123.0, 119.6, 112.9, 47.4. HRMS (ESI) calcd for $C_{21}H_{14}BrN_3O$ m/z): 403.0320, found 403.0341.

1-Bromo-6-butylpyrido[2',1':2,3]imidazo[4,5-c]isoquinolin-5(6H)-one (**8h**)

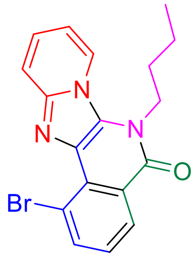
8h

Yellow solid (84% yield), Chemical Formula: $C_{18}H_{16}BrN_3O$. ^{1}H NMR (399 MHz, cdcl$_3$) δ 8.57 (dd, *J* = 8.0, 1.4 Hz, 1H), 8.36 (d, *J* = 7.3 Hz, 1H), 8.09 – 8.02 (m, 1H), 7.80 (d, *J* = 9.3 Hz, 1H), 7.42 – 7.33 (m, 1H), 7.25 (d, *J* = 1.2 Hz, 1H), 7.22 – 7.13 (m, 1H), 6.91 – 6.83 (m, 1H), 4.67 (t, *J* = 7.8 Hz, 2H), 1.92 (t, *J* = 8.0 Hz, 2H), 1.56 (q, *J* = 7.5 Hz, 2H), 1.04 (t, *J* = 7.4 Hz, 3H). ^{13}C NMR (100 MHz, cdcl$_3$) δ 160.6, 141.8, 139.1, 131.1, 130.3, 129.2, 127.2, 126.6, 123.3, 122.6, 120.1, 117.1, 113.3, 43.3, 31.9, 20.0, 13.8. HRMS (ESI) calcd for $C_{18}H_{16}BrN_3O$ m/z): 369.0477, found 369.0480.

1-Bromo-6-(2-morpholinoethyl)pyrido[2',1':2,3]imidazo[4,5-c]isoquinolin-5(6H)-one (**8i**)

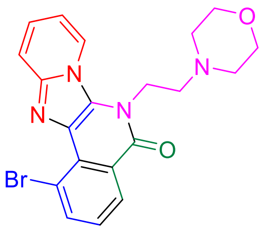

8i

Deep yellow solid (76% yield), Chemical Formula: $C_{20}H_{19}BrN_4O_2$. ^{1}H NMR (399 MHz, cdcl$_3$) δ 8.57 (dd, J = 8.0, 1.4 Hz, 1H), 8.36 (d, J = 7.3 Hz, 1H), 8.09 – 8.02 (m, 1H), 7.80 (d, J = 9.3 Hz, 1H), 7.42 – 7.33 (m, 1H), 7.22 – 7.13 (m, 1H), 6.91 – 6.83 (m, 1H), 4.45 (ddd, J = 14.0, 7.3, 4.5 Hz, 2H), 3.61 – 3.50 (m, 3H), 3.45 (d, J = 7.1 Hz, 4H), 2.62 – 2.52 (m, 3H), 2.29 (ddd, J = 12.7, 7.1, 4.1 Hz, 3H). ^{13}C NMR (125 MHz, Common NMR Solvents) δ 149.0, 132.7, 123.3, 118.2, 118.0, 117.0, 116.3, 115.9, 115.5, 115.3, 114.1, 107.0, 111.1, 100.3, 54.6, 43.3, 41.8, 35.3.

6-Butyl-9-chloropyrido[2',1':2,3]imidazo[4,5-c]isoquinolin-5(6H)-one (**8l**)

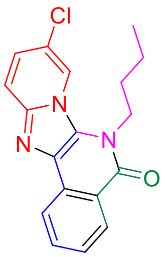

8l

White solid (68% yield), Chemical Formula: $C_{18}H_{16}ClN_3O$. ^{1}H NMR (399 MHz, cdcl$_3$) δ 8.52 (dd, J = 14.1, 8.1 Hz, 2H), 8.32 (d, J = 7.1 Hz, 1H), 7.86 – 7.77 (m, 1H), 7.58 (t, J = 7.5 Hz, 1H), 7.32 – 7.22 (m, 1H), 7.25 (s, 2H), 7.25 (s, 2H), 6.82 (t, J = 7.2 Hz, 1H), 4.65 (t, J = 7.9 Hz, 2H), 1.95 – 1.86 (m, 2H), 1.64 – 1.55 (m, 2H), 1.04 (t, J = 7.4 Hz, 3H). ^{13}C NMR (100 MHz, cdcl$_3$) δ 133.0, 129.2, 127.5, 124.8, 122.5, 121.5, 113.0, 43.1, 32.2, 20.0, 13.8. HRMS (ESI) calcd for $C_{18}H_{16}ClN_3O$: 325.0982, found 325.0915.

9-Bromo-6-butylpyrido[2',1':2,3]imidazo[4,5-c]isoquinolin-5(6H)-one (**8m**)

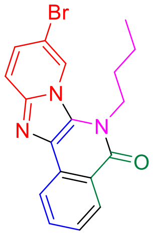

8m

White solid (80% yield), Chemical Formula: $C_{18}H_{16}BrN_3O$. ^{1}H NMR (399 MHz, cdcl$_3$) δ 8.51 (d, J = 8.7 Hz, 2H), 8.39 (d, J = 7.9 Hz, 1H), 7.81 (t, J = 7.7 Hz, 1H), 7.64 – 7.50 (m, 2H), 7.23 (dd, J = 13.4, 3.7 Hz, 2H), 4.63 (t, J = 7.8 Hz, 2H), 1.91 (q, J = 7.8 Hz, 2H), 1.12 – 1.03 (m, 3H). ^{13}C NMR (100 MHz, cdcl$_3$) δ 133.1, 129.4, 127.5, 126.7, 122.6, 121.9, 119.5, 42.6, 29.7, 19.9, 13.8. HRMS (ESI) calcd for $C_{18}H_{16}BrN_3O$ m/z): 369.0477, found 369.0480.

10-Bromo-6-butylpyrido[2',1':2,3]imidazo[4,5-c]isoquinolin-5(6H)-one (**8n**)

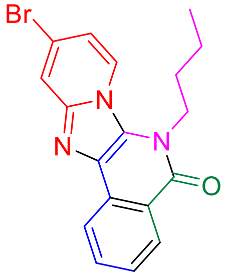

8n

White solid (84% yield), Chemical Formula: $C_{18}H_{16}BrN_3O$.^{1}H NMR (399 MHz, cdcl$_3$) δ 8.55 – 8.48 (m, 1H), 8.45 – 8.32 (m, 2H), 7.85 – 7.76 (m, 1H), 7.75 – 7.68 (m, 1H), 7.61 – 7.52 (m, 1H), 7.23 – 7.14 (m, 1H), 4.67 (t, J = 7.9 Hz, 2H), 1.92 (t, J = 7.7 Hz, 2H), 1.58 (h, J = 7.4 Hz, 2H), 1.16 (s, 2H), 1.08 – 1.00 (m, 3H). ^{13}C NMR (100 MHz, cdcl$_3$) δ 167.2, 147.2, 143.5, 130.3, 129.4 126.7, 122.4, 121.9, 120.1, 119.9, 117.4, 111.6, 109.2, 48.2, 30.4, 20.2, 13.7. HRMS (ESI) calcd for $C_{18}H_{16}BrN_3O$ m/z): 369.0477, found 369.0480.

6-Isopropylpyrido[2',1':2,3]imidazo[4,5-c]isoquinolin-5(6H)-one (**8p**)

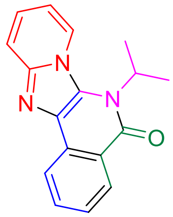

8p

White solid (72% yield), Chemical Formula: $C_{17}H_{15}N_3O$.^{1}H NMR (399 MHz, cdcl$_3$) δ 8.51 (d, 1H), 8.40 (d, 1H), 8.37 (d, 1H), 7.84 – 7.81 (m, 1H), 7.74 – 7.72 (m, 1H), 7.57 – 7.54 (m, 1H), 7.19 (t, 1H), 6.86 (t, J = 7.7 Hz, 1H), 4.93 (m, *1*H), 1.87 (d, 6H). ^{13}C NMR (100 MHz, cdcl$_3$) δ 143.1, 136.3, 130.21, 128.5, 128.4, 126.1, 122.4, 117.6, 115.4, 112.1, 51.2, 24.2. HRMS (ESI) calcd for $C_{17}H_{15}N_3O$. m/z): 277.1215, found 277.1205.

6-Benzylpyrido[2',1':2,3]imidazo[4,5-c]isoquinolin-5(6H)-one (**8q**)

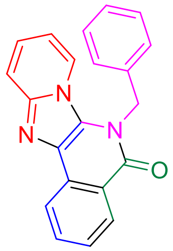

8q

Light yellow solid (78% yield), Chemical Formula: $C_{21}H_{15}N_3O$. ^{1}H NMR (399 MHz, cdcl$_3$) δ 8.57 (d, J = 8.0 Hz, 1H), 8.48 (dd, J = 16.3, 8.0 Hz, 1H), 8.37 (d, J = 8.0 Hz, 1H), 8.26 (d, J = 7.8 Hz, 1H), 8.19 – 8.12 (m, 1H), 7.90 – 7.77 (m, 1H), 7.72 – 7.60 (m, 1H), 7.62 – 7.53 (m, 1H), 7.40 – 7.22 (m, 3H), 7.12 – 7.04 (m, 1H), 6.89 – 6.82 (m, 1H), 6.60 (t, J = 7.0 Hz, 1H), 4.62 (d, J = 7.9 Hz, 1H). ^{13}C NMR (100 MHz, cdcl$_3$) δ 133.4, 133.1, 129.6, 129.4, 129.4, 127.9, 127.4, 127.3, 125.5, 123.2, 122.9, 121.9, 117.6, 114.6, 46.9. HRMS (ESI) calcd for $C_{21}H_{15}N_3O$ m/z): 325.1215, found 325.1232.

6-Butylpyrido[2',1':2,3]imidazo[4,5-c]isoquinolin-5(6H)-one (**8r**)

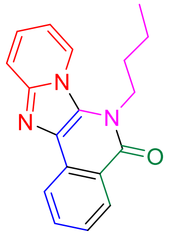

8r

Deep yellow solid(80% yield), Chemical Formula: $C_{18}H_{17}N_3O$. ^{1}H NMR (399 MHz, cdcl$_3$) δ 8.55 – 8.48 (m, 1H), 8.45 – 8.32 (m, 2H), 7.85 – 7.76 (m, 1H), 7.75 – 7.68 (m, 1H), 7.61 – 7.52 (m, 1H), 7.28 – 7.14 (m, 1H), 6.92 – 6.83 (m, 1H), 4.67 (t, J = 7.9 Hz, 2H), 1.92 (t, J = 7.7 Hz, 2H), 1.58 (h, J = 7.4 Hz, 2H), 1.08 – 1.00 (m, 3H). ^{13}C NMR (100 MHz, cdcl$_3$) δ 161.4, 143.0, 133.0, 131.6, 129.3, 127.2, 124.1, 123.5, 122.9, 122.8, 121.9, 119.2, 112.9, 42.8, 32.2, 20.0, 13.8. HRMS (ESI) calcd for $C_{18}H_{17}N_3O$ m/z): 291.1372, found 291.1369.

6-(2-Morpholinoethyl)pyrido[2',1':2,3]imidazo[4,5-c]isoquinolin-5(6H)-one (**8s**)

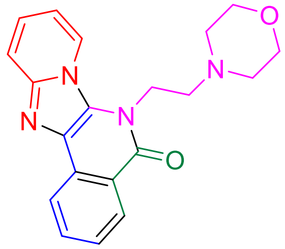

8s

Yellow solid (60% yield), Chemical Formula: $C_{20}H_{20}N_4O_2$. ^{1}H NMR (399 MHz, cdcl$_3$) δ 8.77 (s, 1H), 8.50 (d, J = 8.2 Hz, 1H), 8.42 (d, J = 8.0 Hz, 1H), 7.82 (t, J = 7.5 Hz, 1H), 7.72 (d, J = 9.4 Hz, 1H), 7.57 (t, J = 7.7 Hz, 1H), 7.25 (s, 2H), 6.89 (t, J = 6.7 Hz, 1H), 4.82 (s, 2H), 3.72 (s, 5H), 3.63 (s, 2H), 2.95 (s, 2H), 2.88 (s, 3H). ^{13}C NMR (100 MHz, cdcl$_3$) δ 168.1, 149.3, 143.2, 132.7, 131.6, 127.0, 126.4, 123.6, 117.1, 111.8, 111.2, 108.3, 66.6, 56.5, 52.1, 44.2.

^{1}H NMR, ^{13}C NMR spectra of products **6a** and **8**

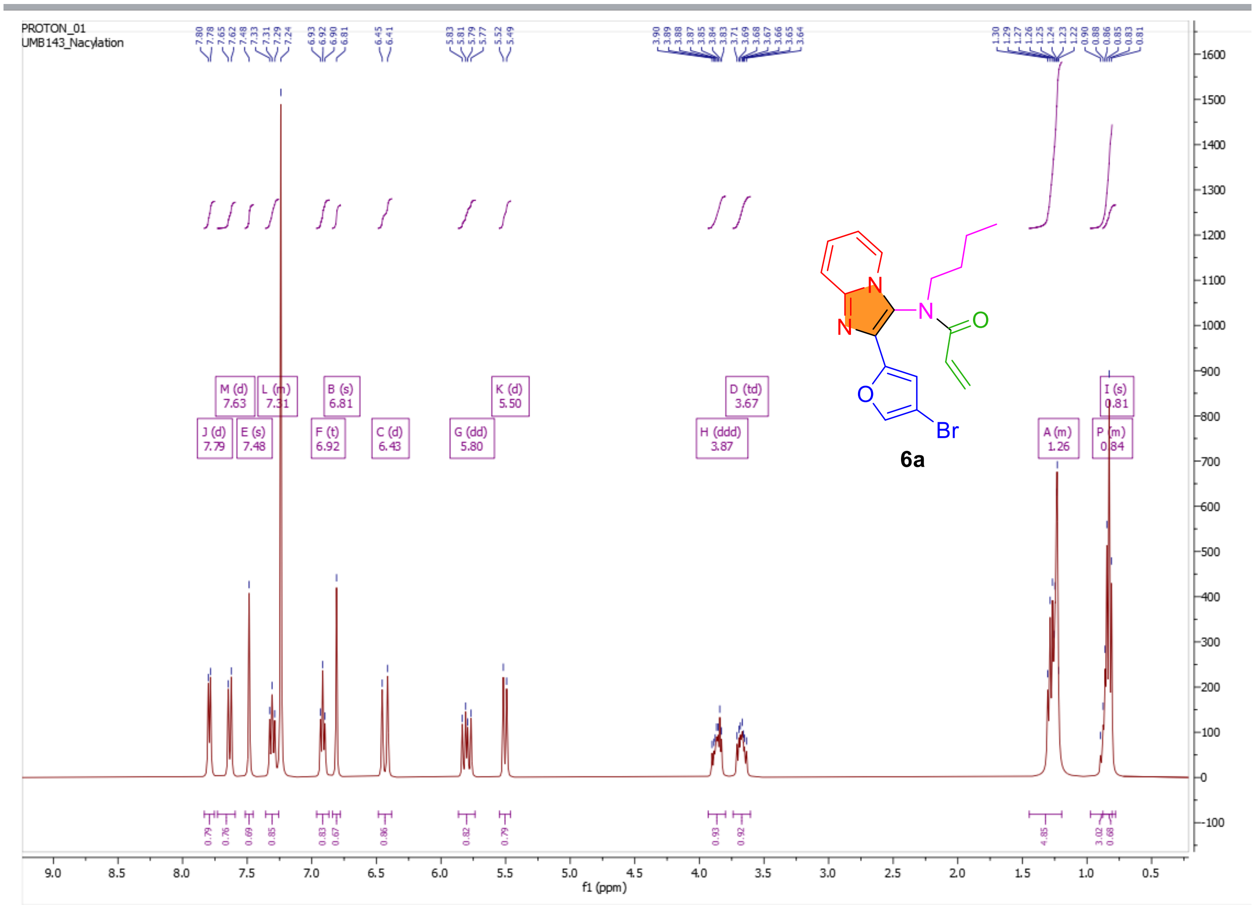

^{1}H NMR of **6a**

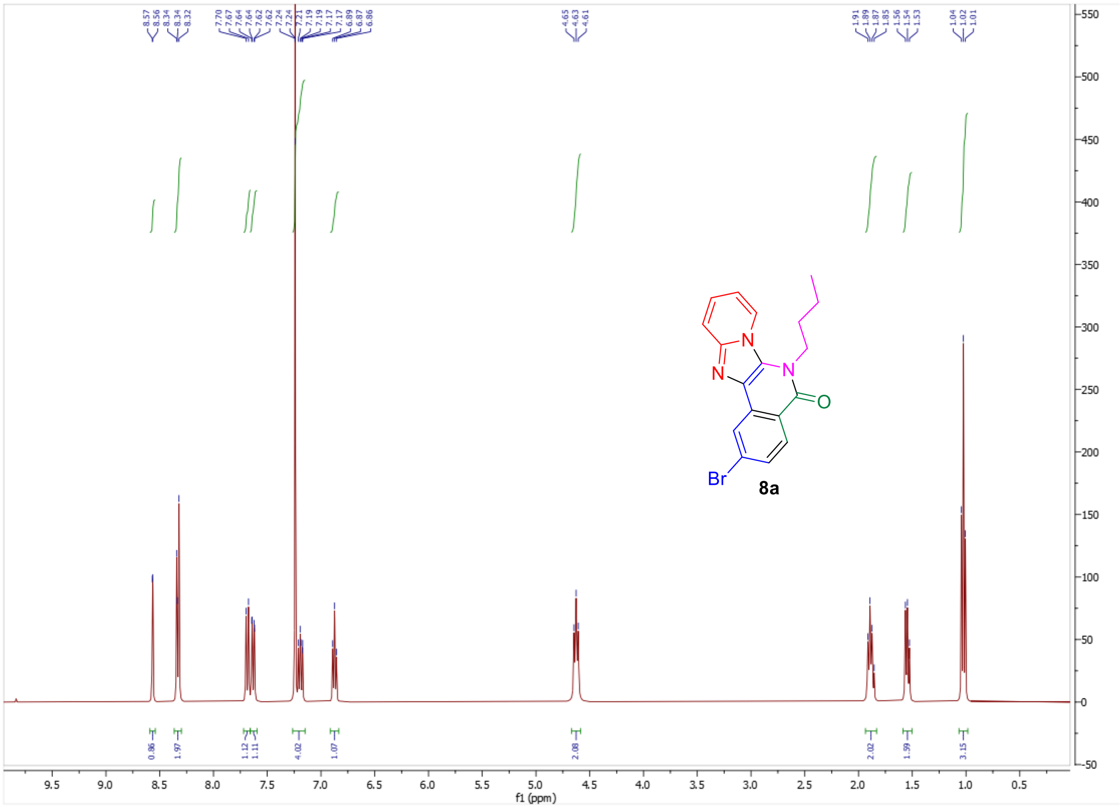

^{1}H NMR of **8a**

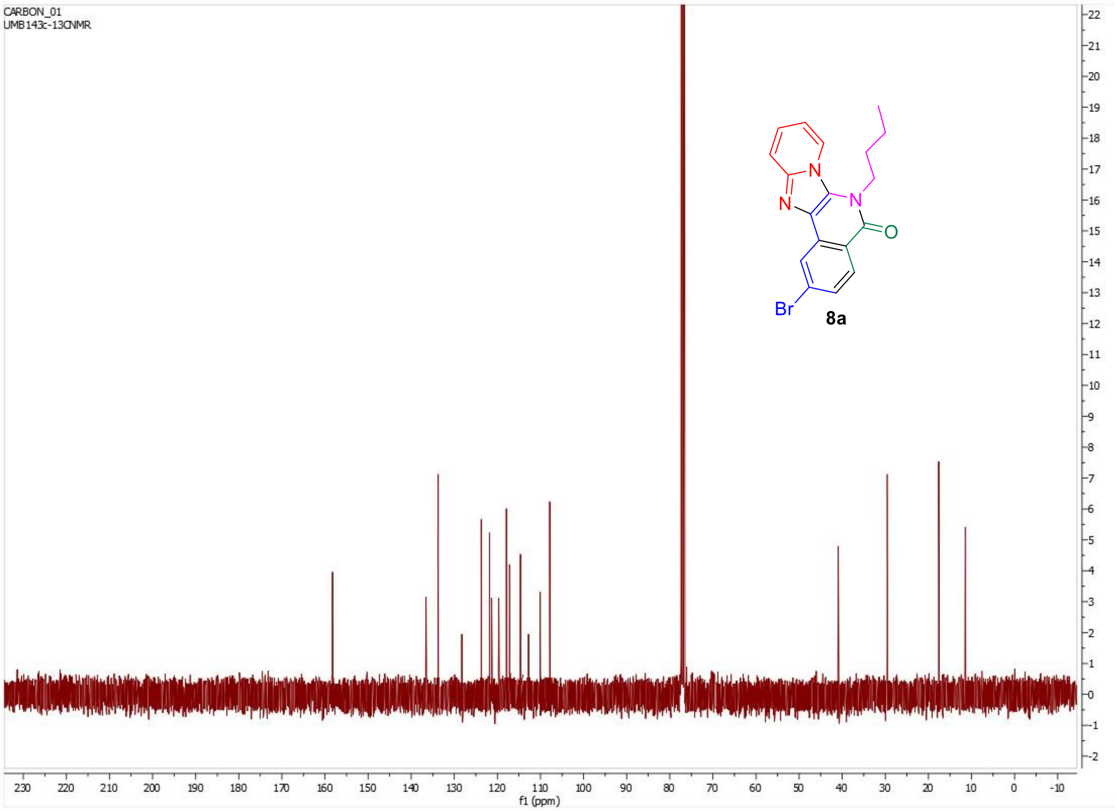

^{13}C NMR of **8a**

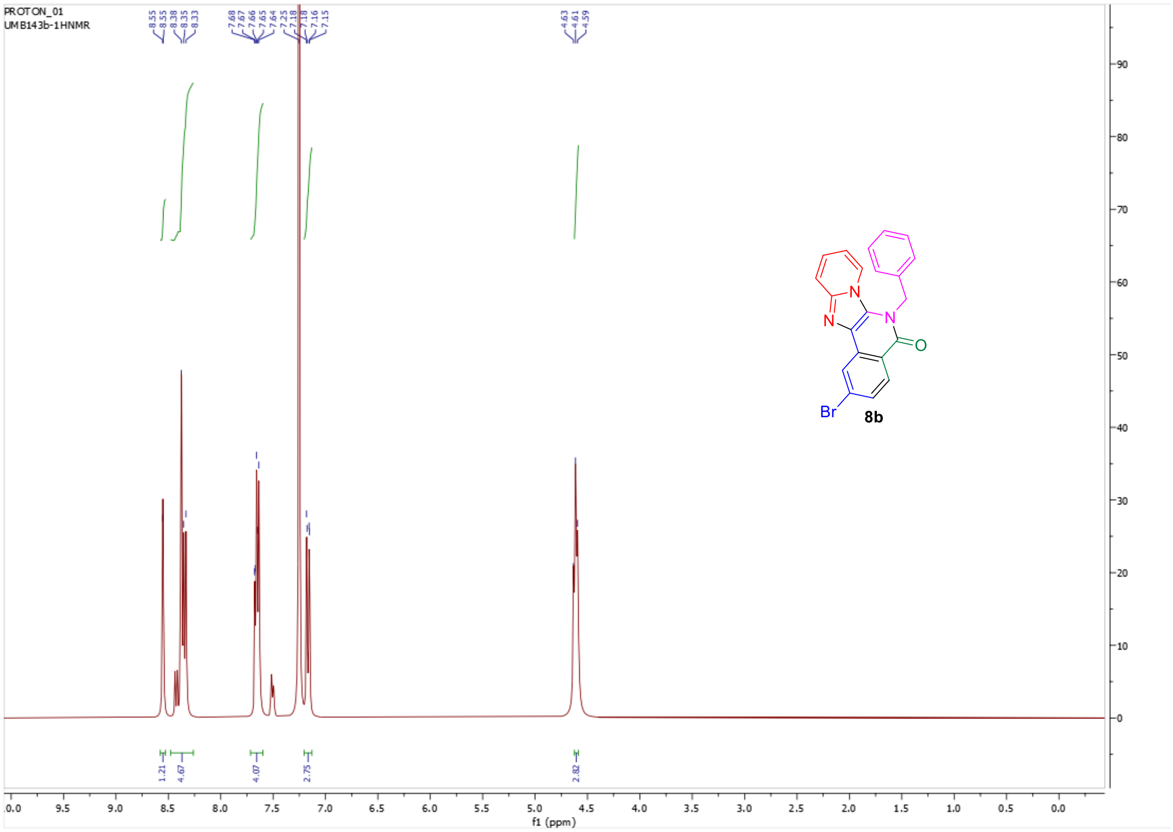

^{1}H NMR of **8b**

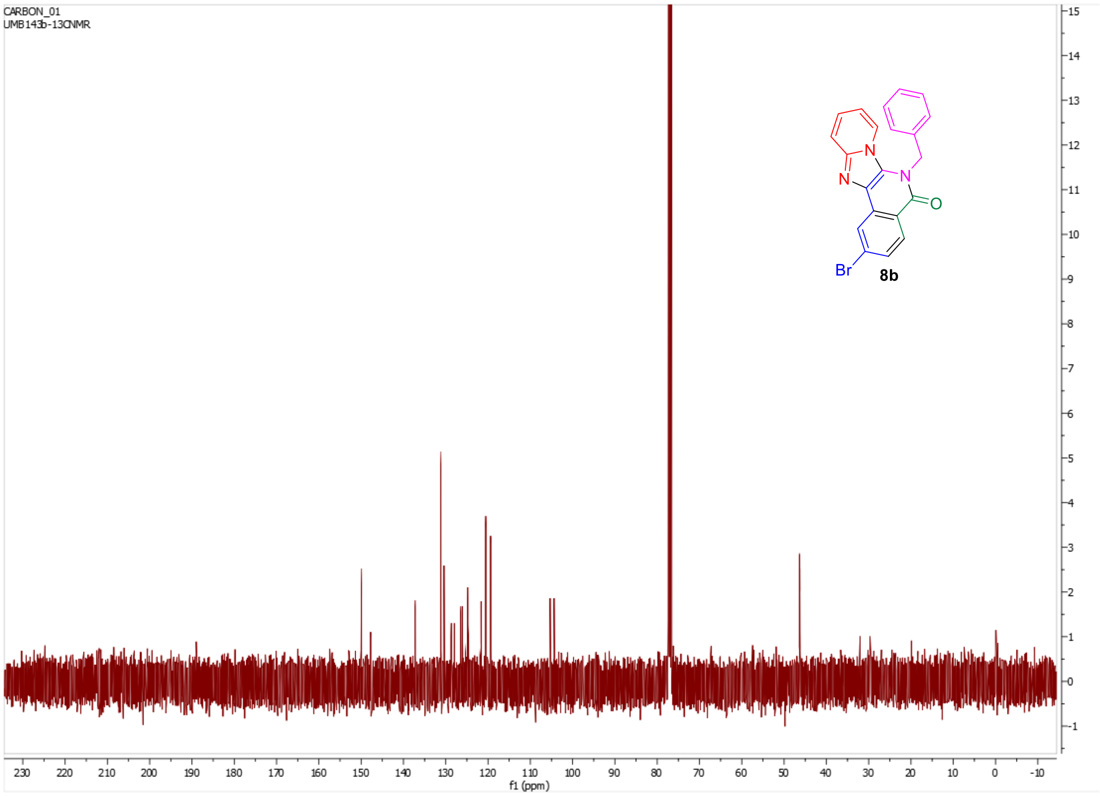

^{13}C NMR of **8b**

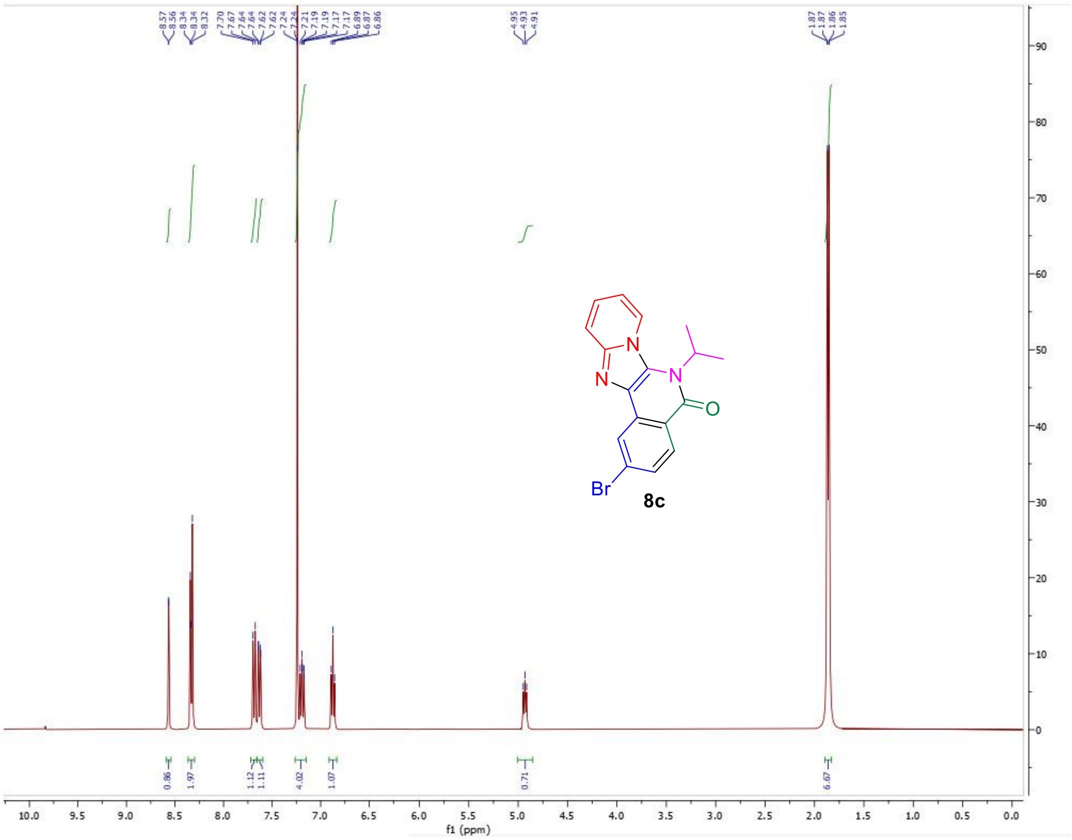

^{1}H NMR of **8c**

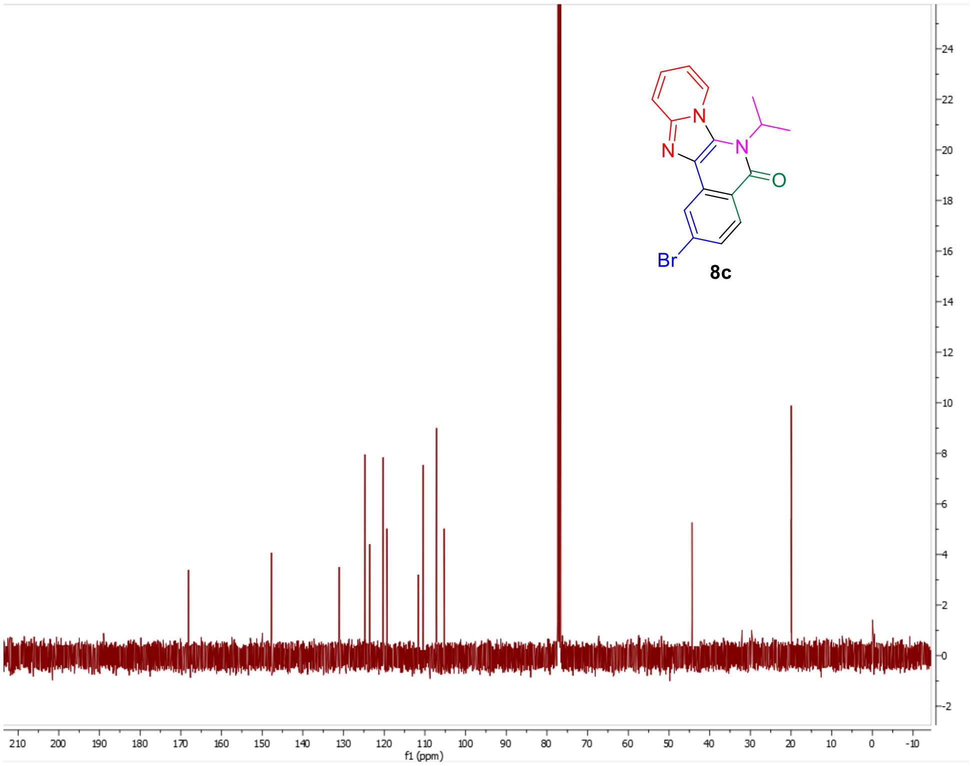

^{13}C NMR of **8c**

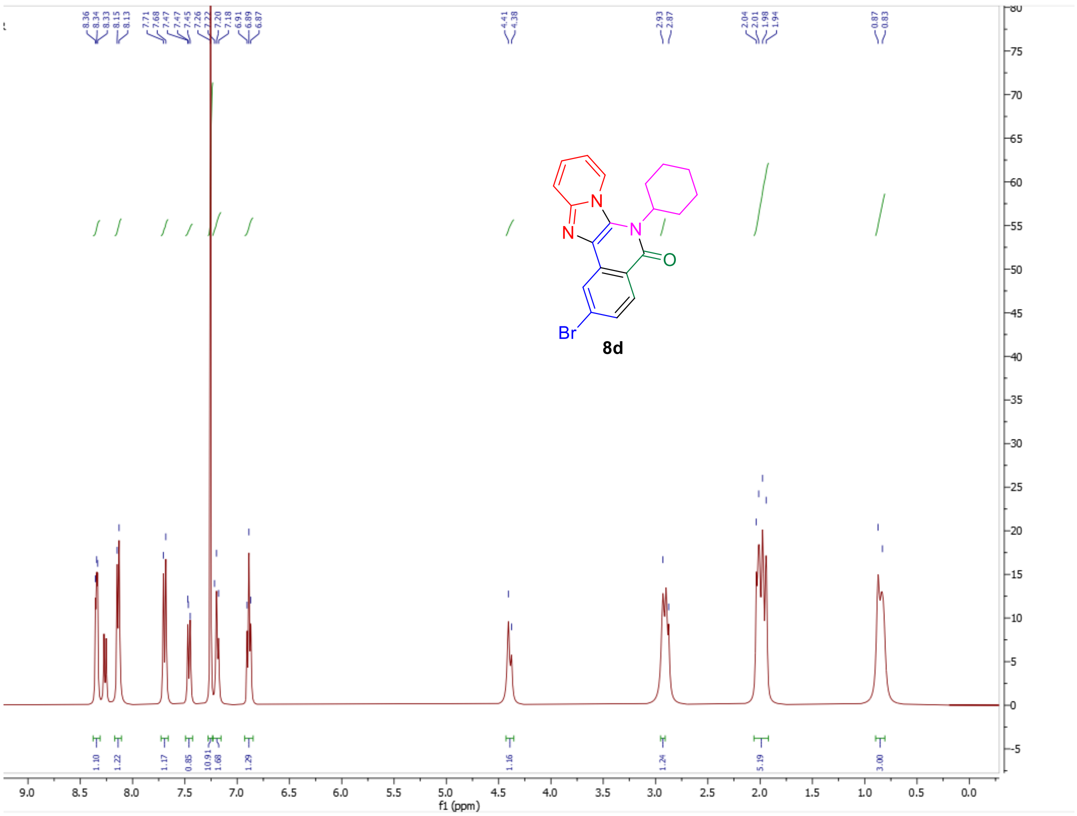

^{1}H NMR of **8d**

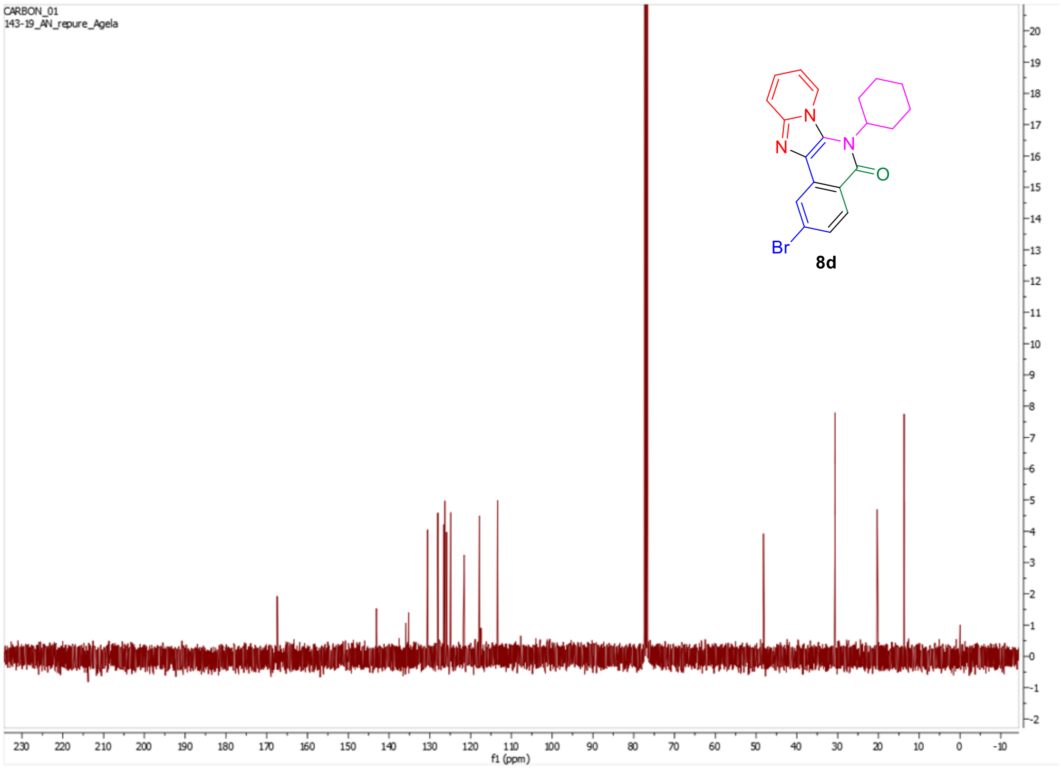

^{13}C NMR of **8d**

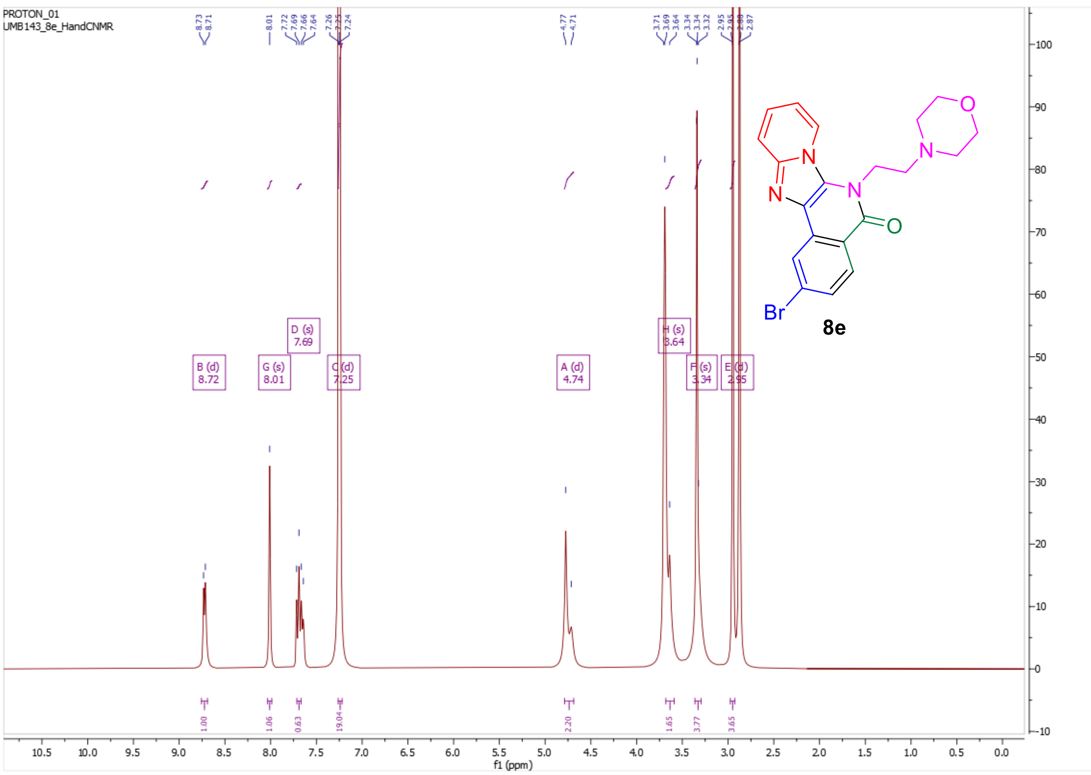

^{1}H NMR of **8e**

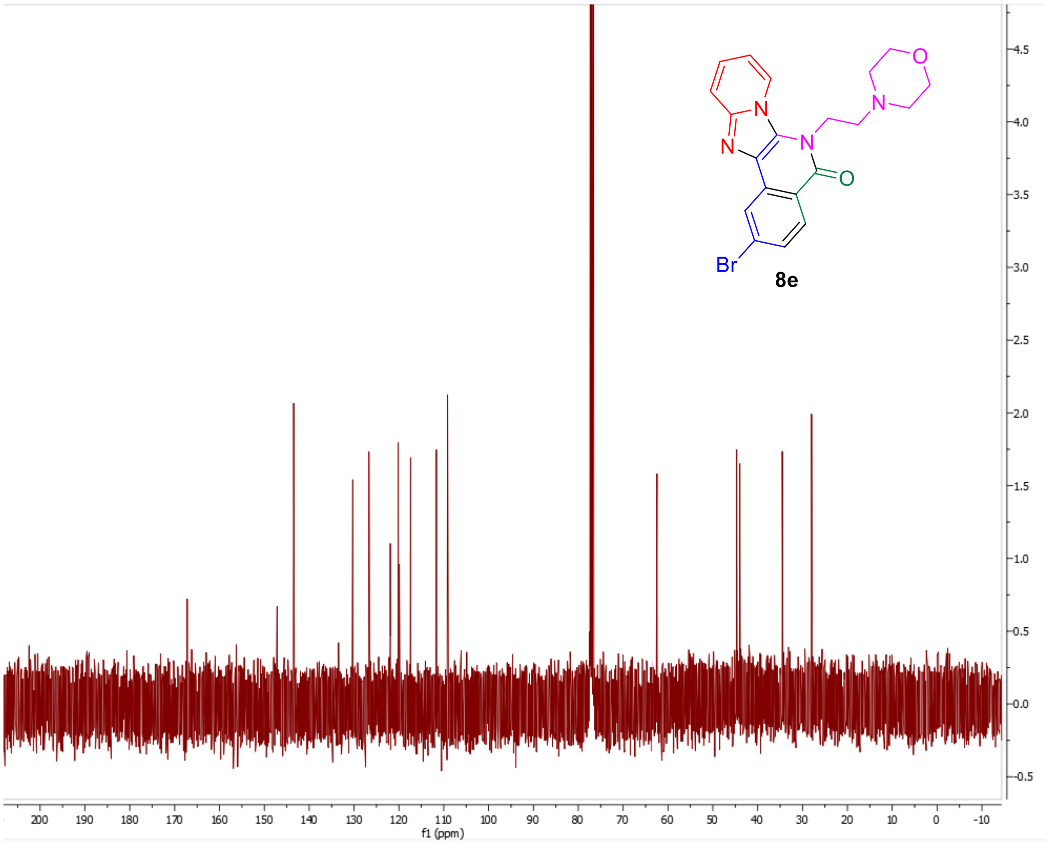

^{13}C NMR of **8e**

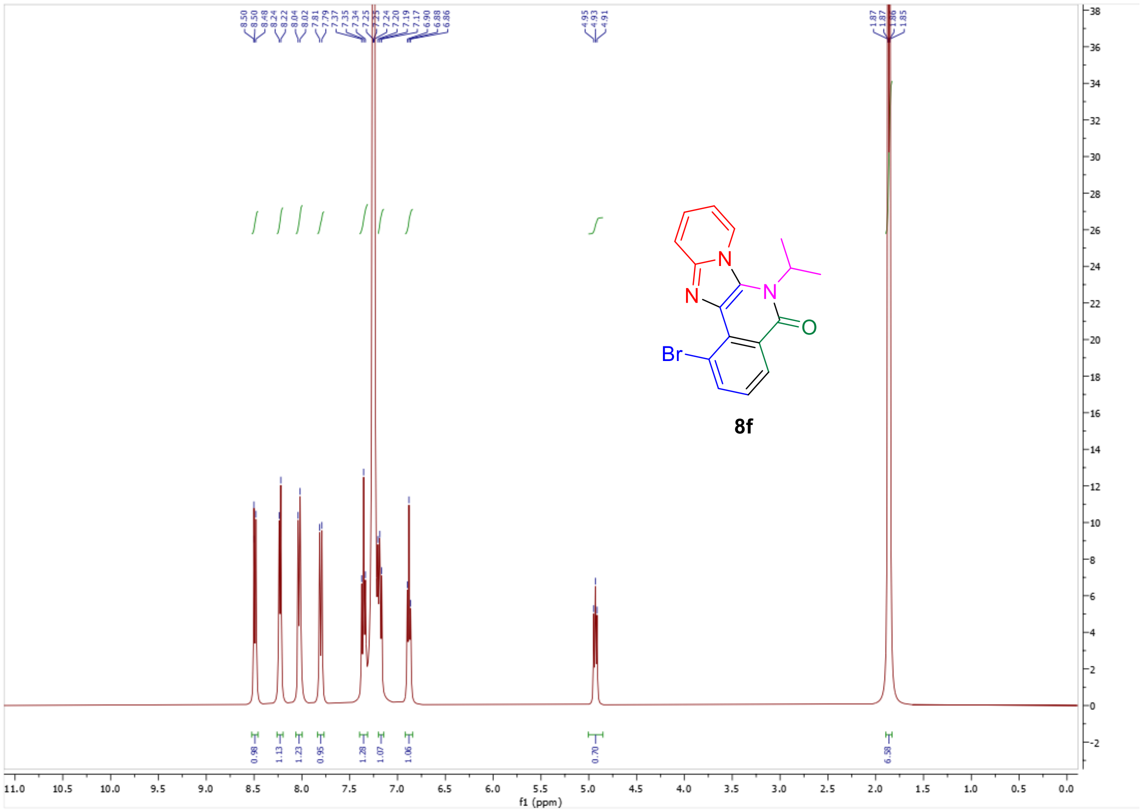

^{1}H NMR of **8f**

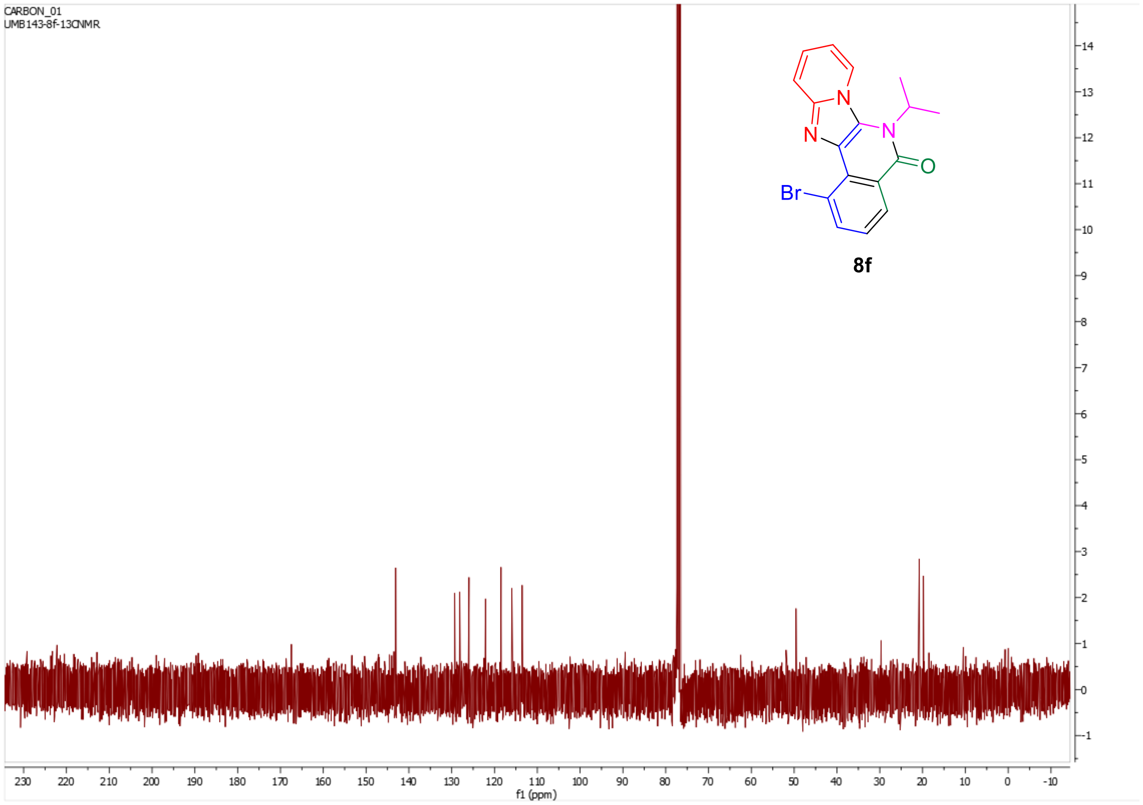

^{13}C NMR of **8f**

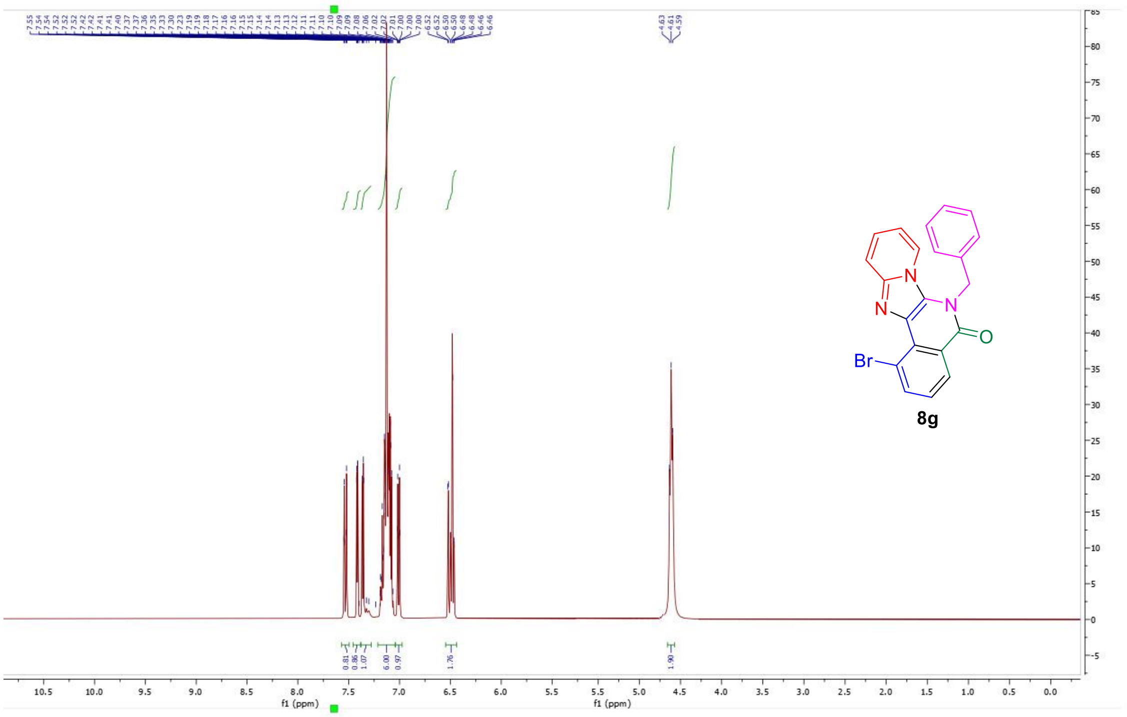

^{1}H NMR of **8g**

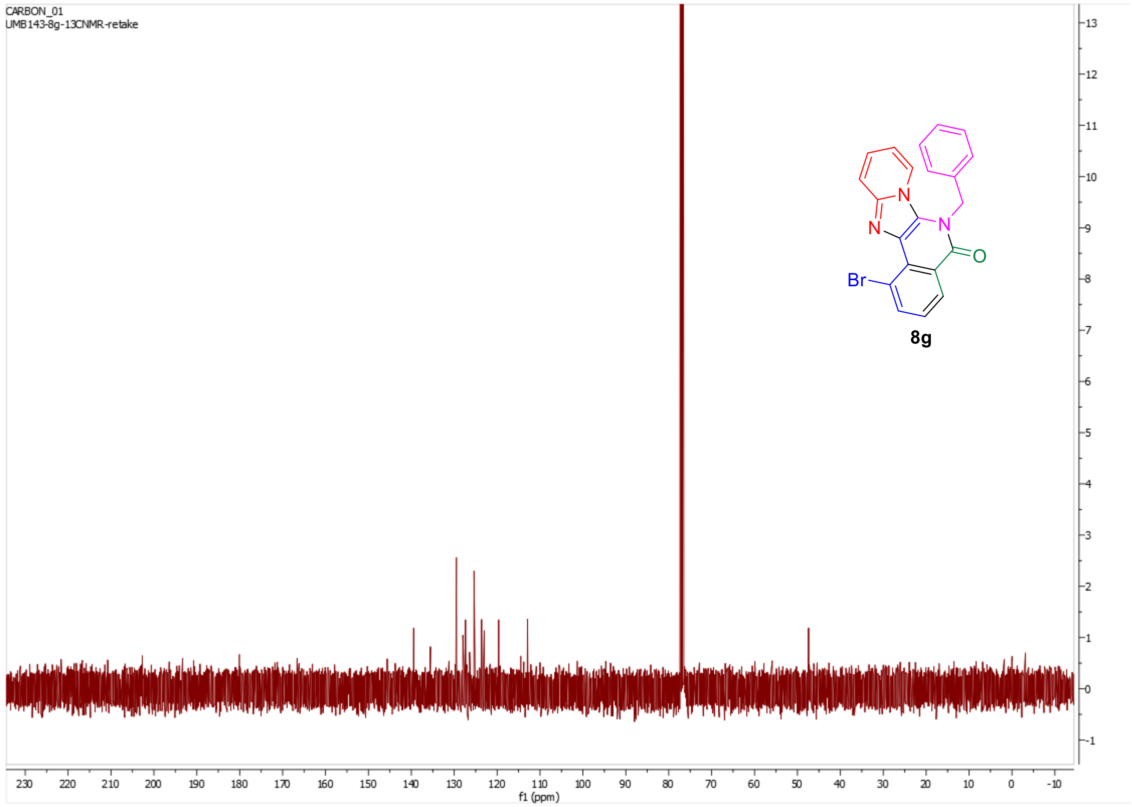

^{13}C NMR of 8g

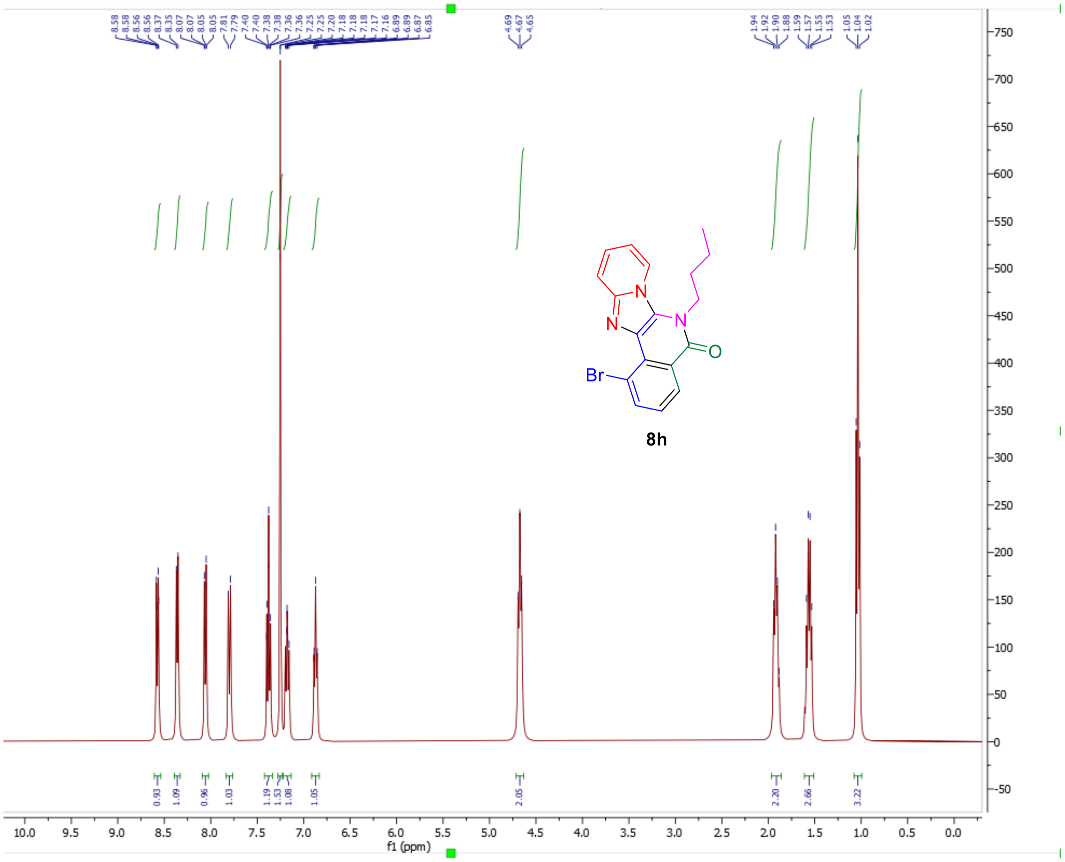

^{1}H NMR of 143

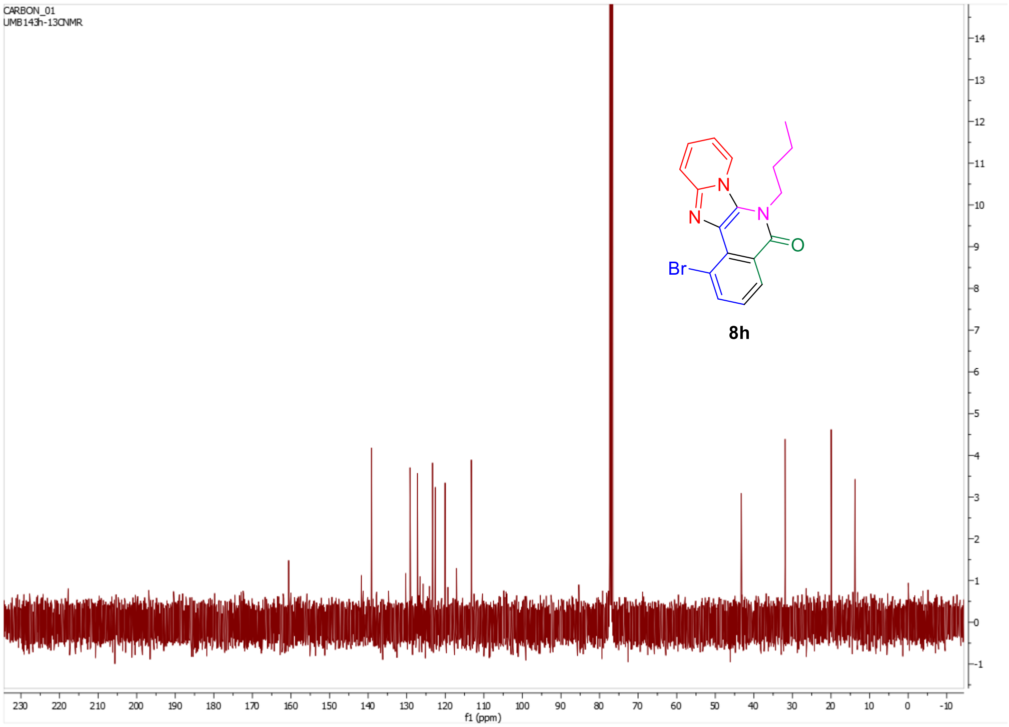

^{13}C NMR of **8h**

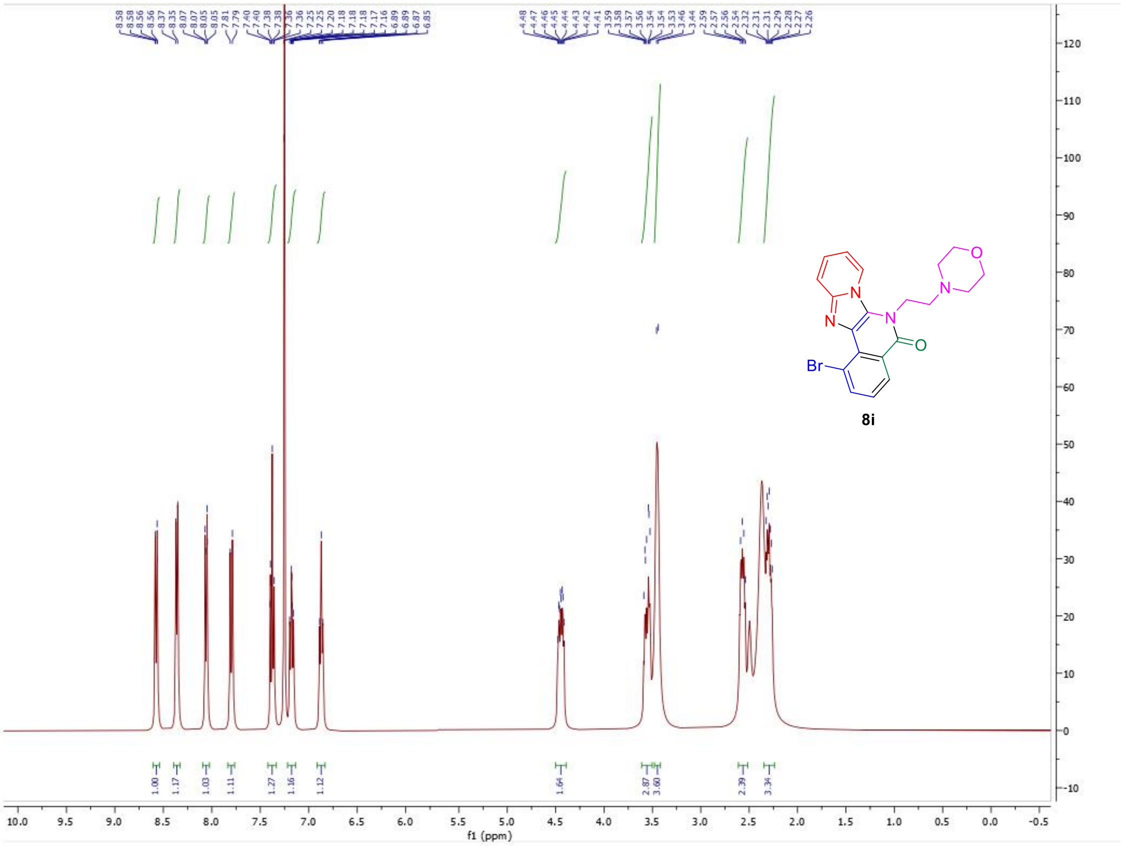

^{1}H NMR of **8i**

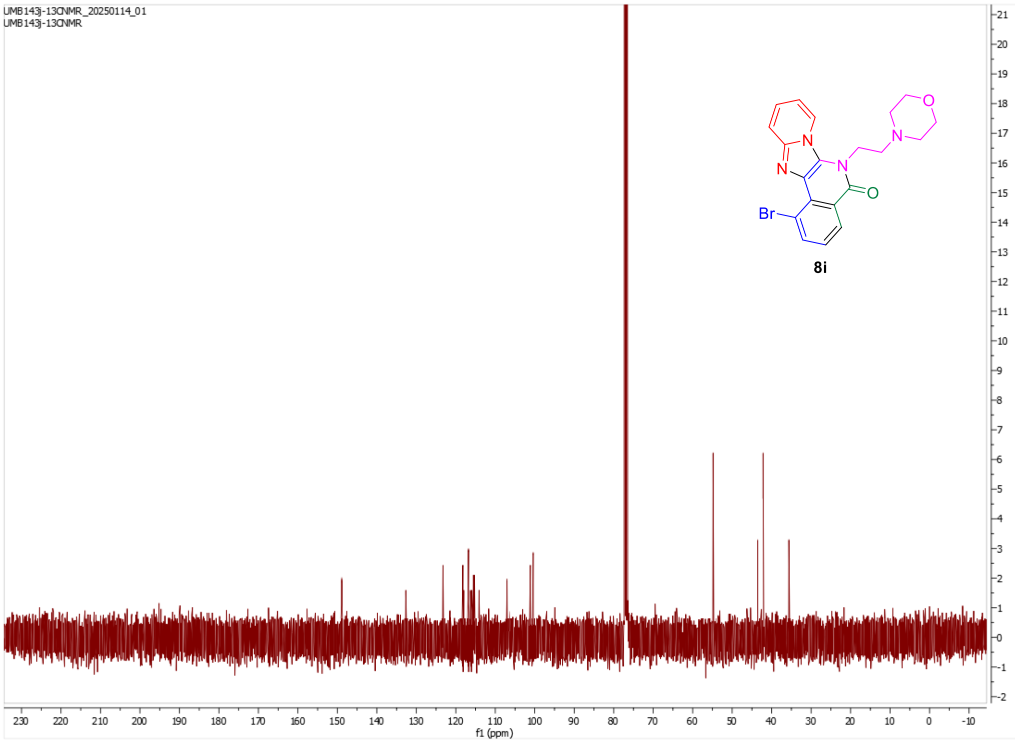

^{13}C NMR of **8i**

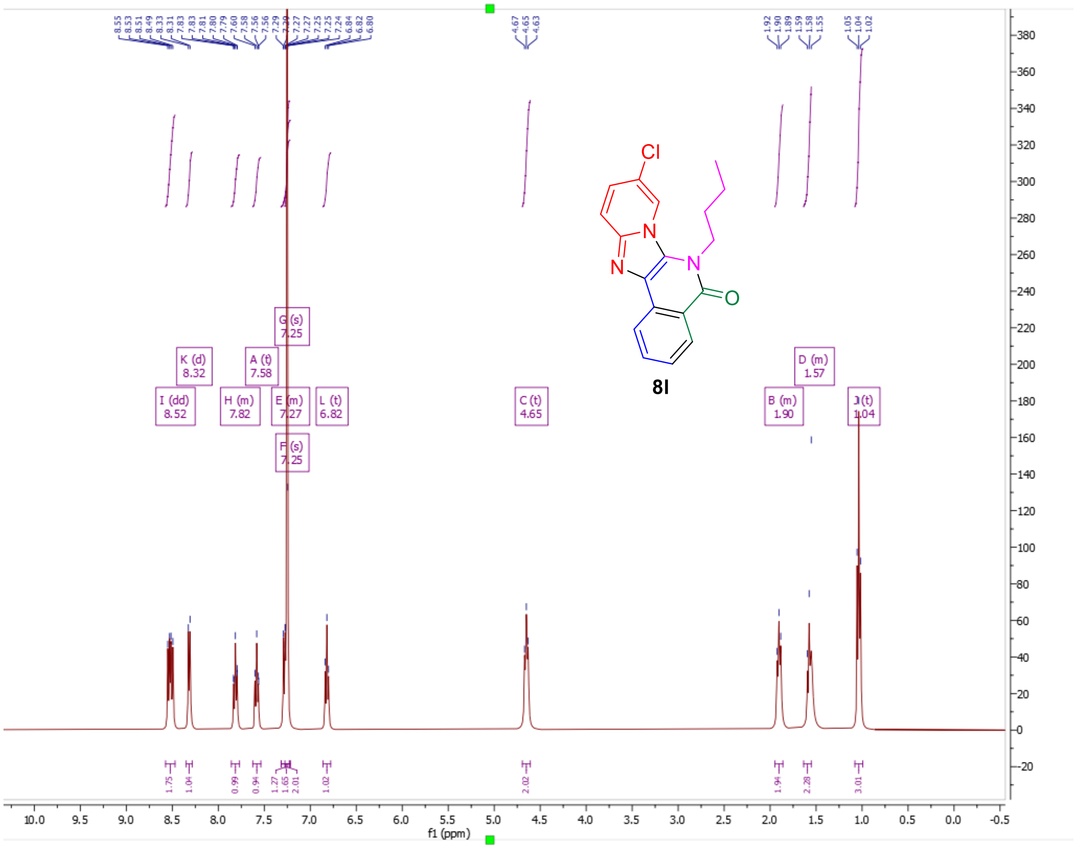

^{1}H NMR of **8l**

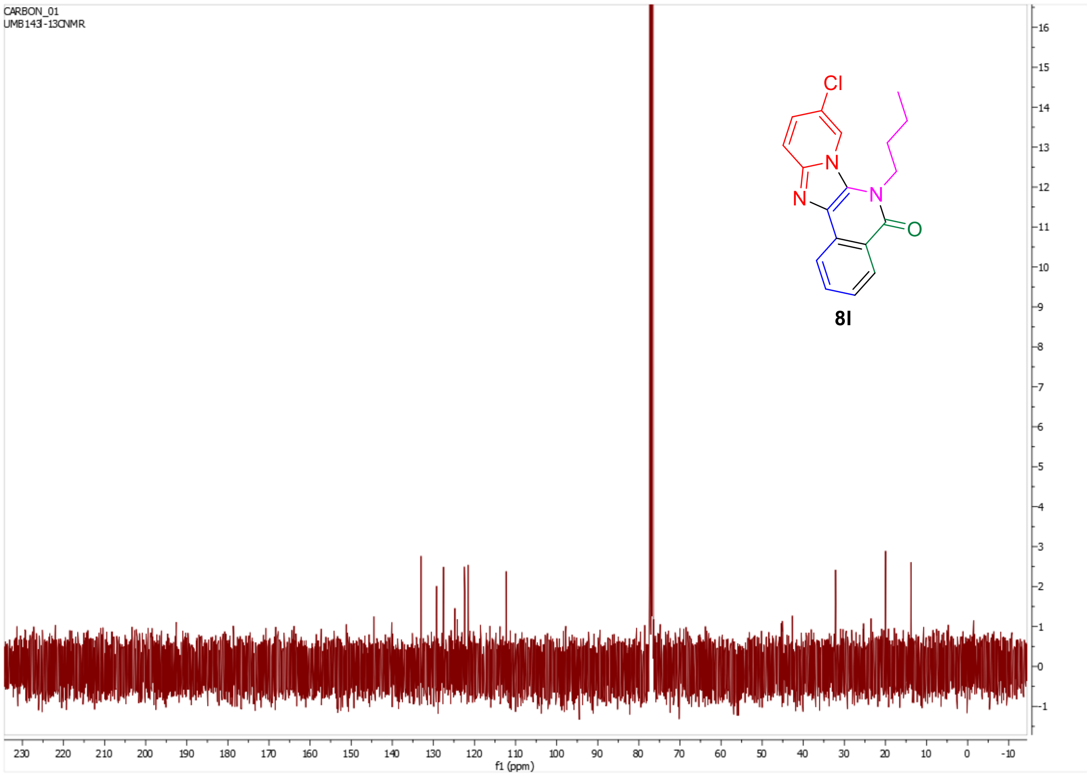

^{13}C NMR of **8l**

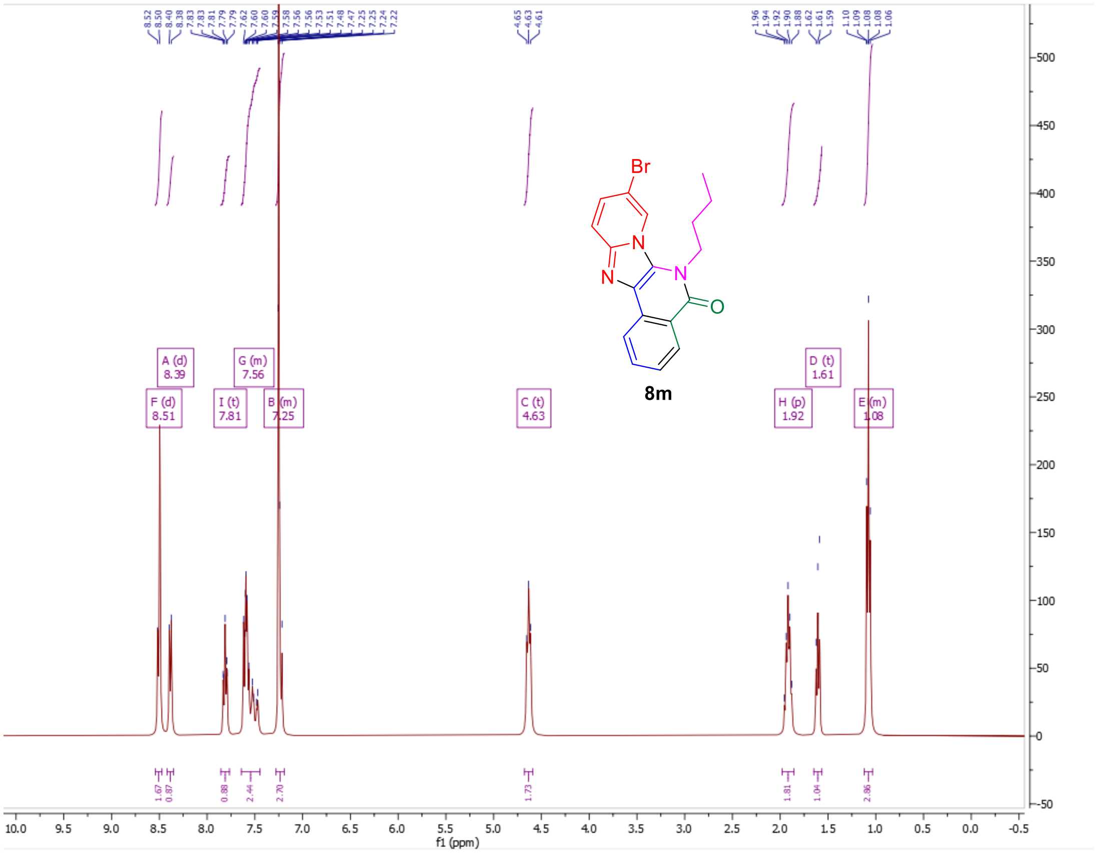

^{1}H NMR of **8m**

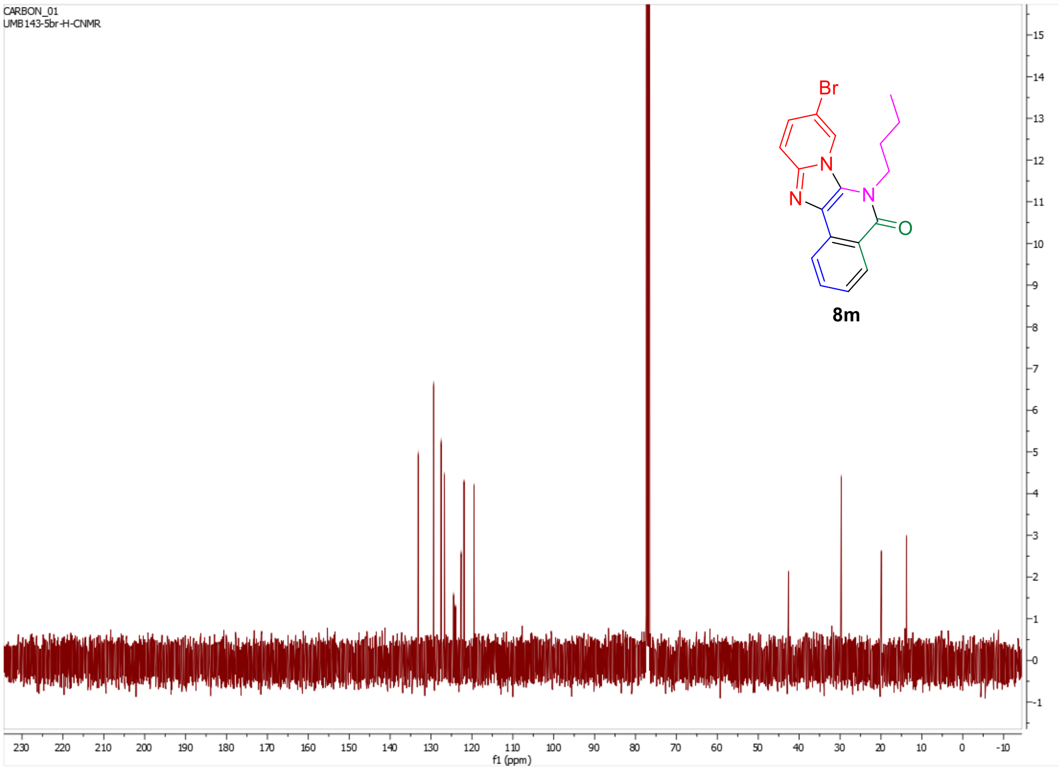

^{13}C NMR of **8m**

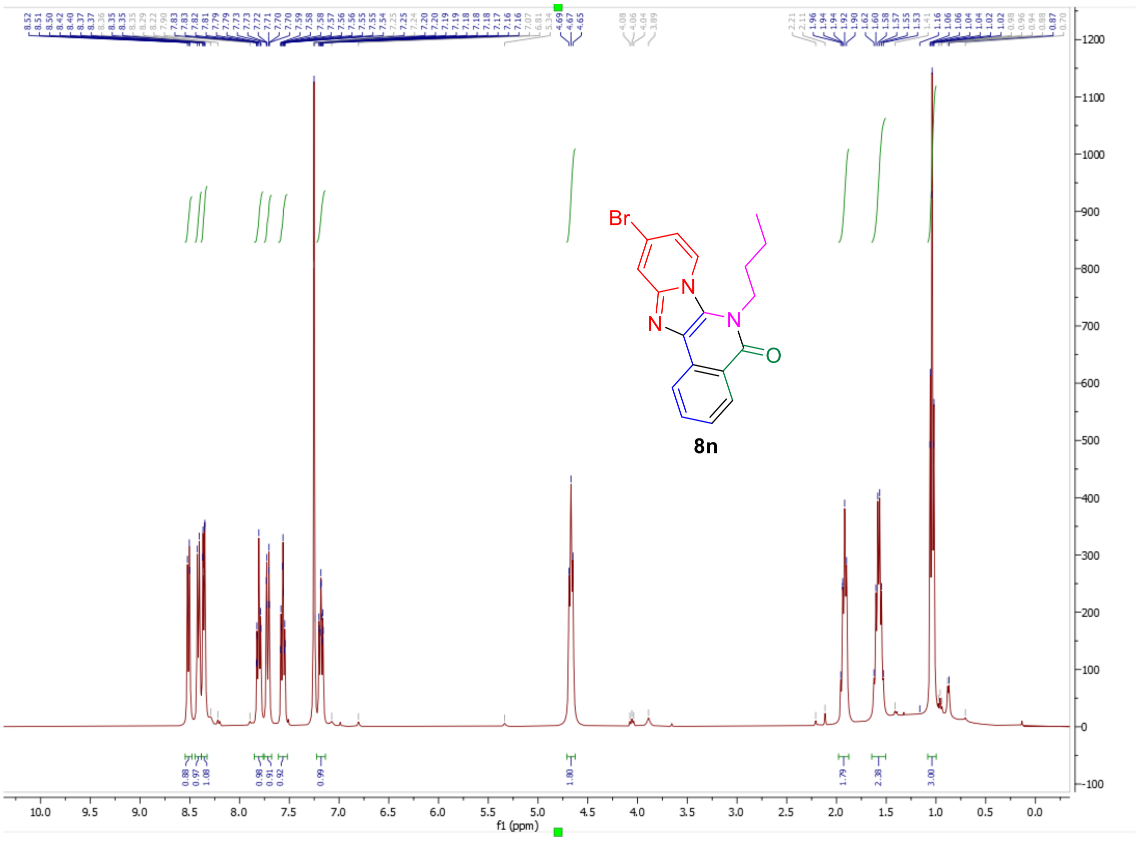

^{1}H NMR of **8n**

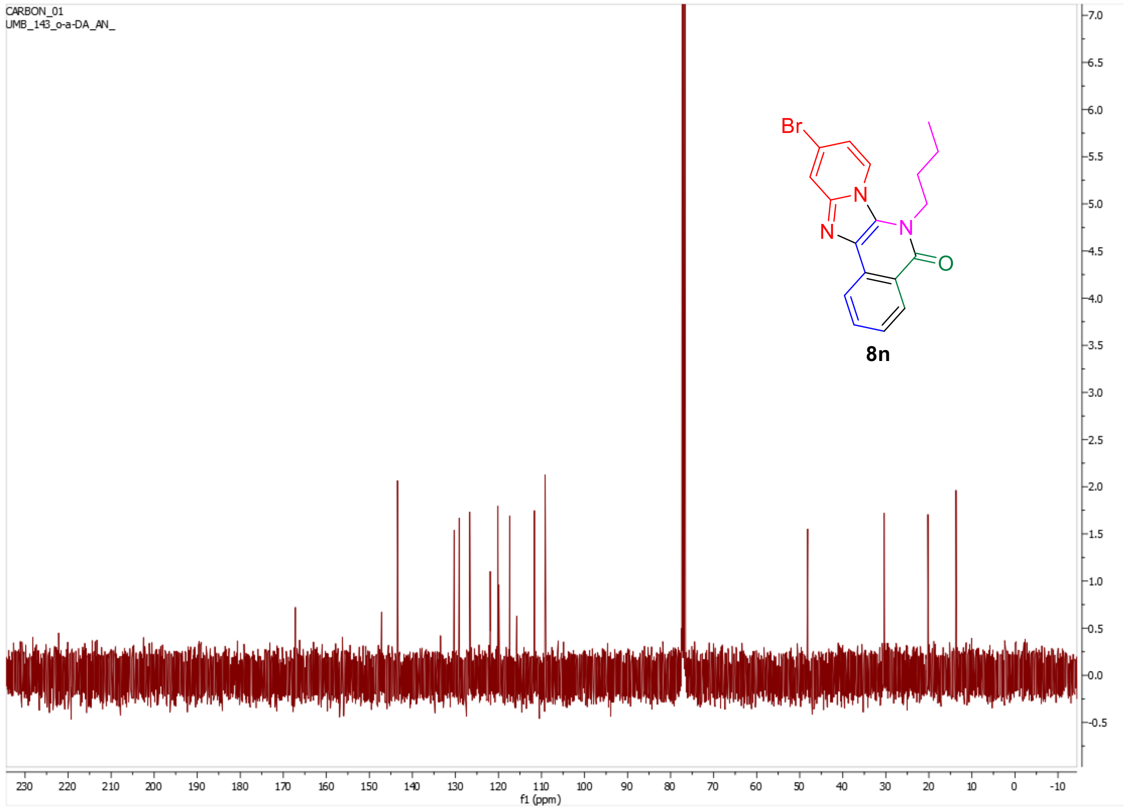

^{13}C NMR of **8n**

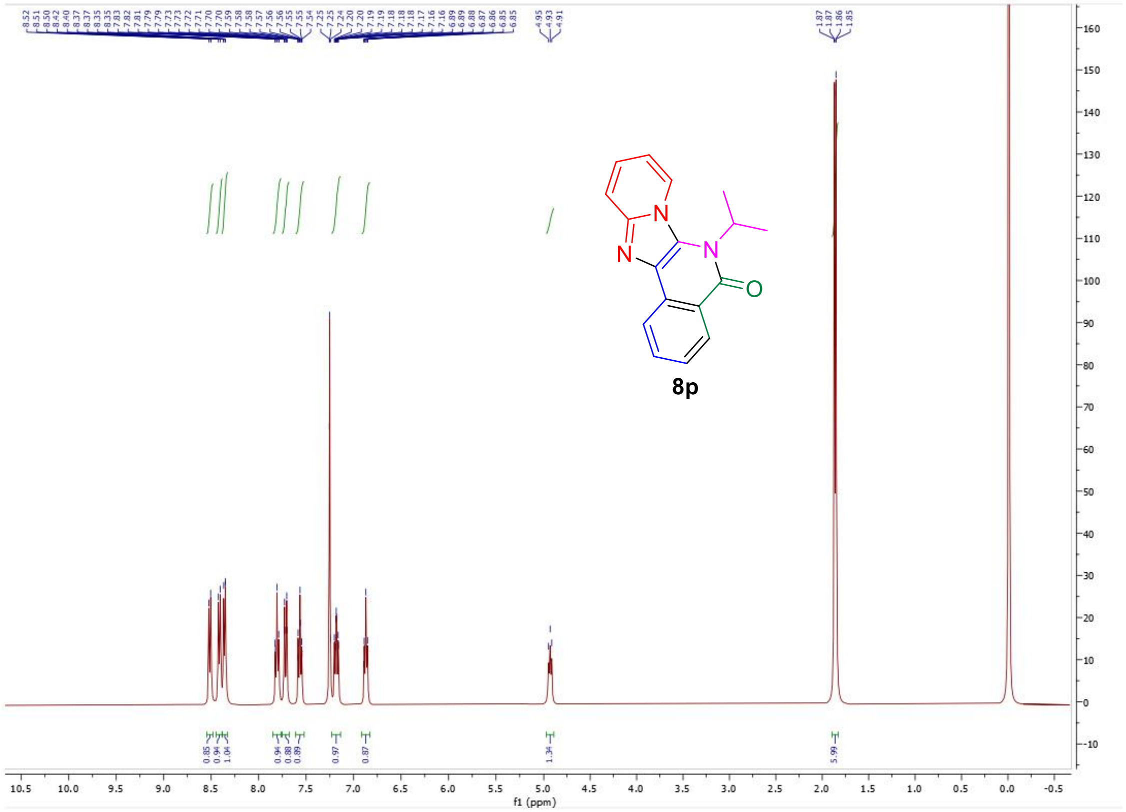

^{1}H NMR of **8p**

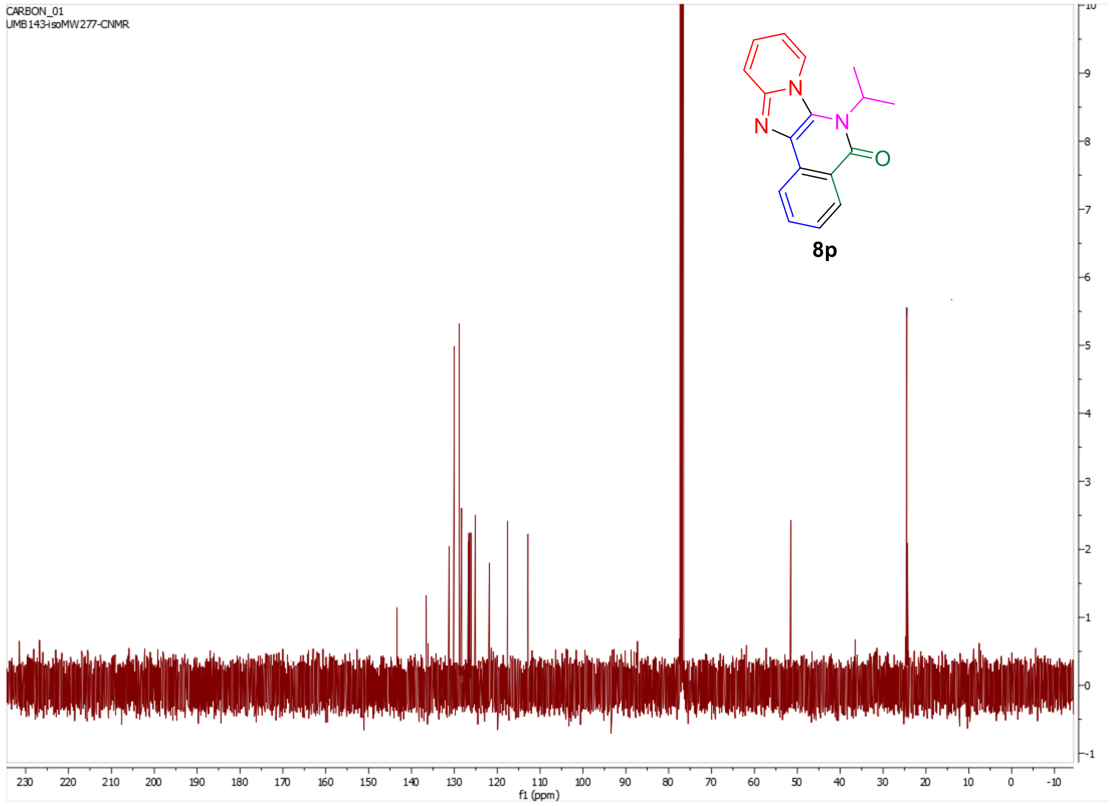

^{13}C NMR of **8p**

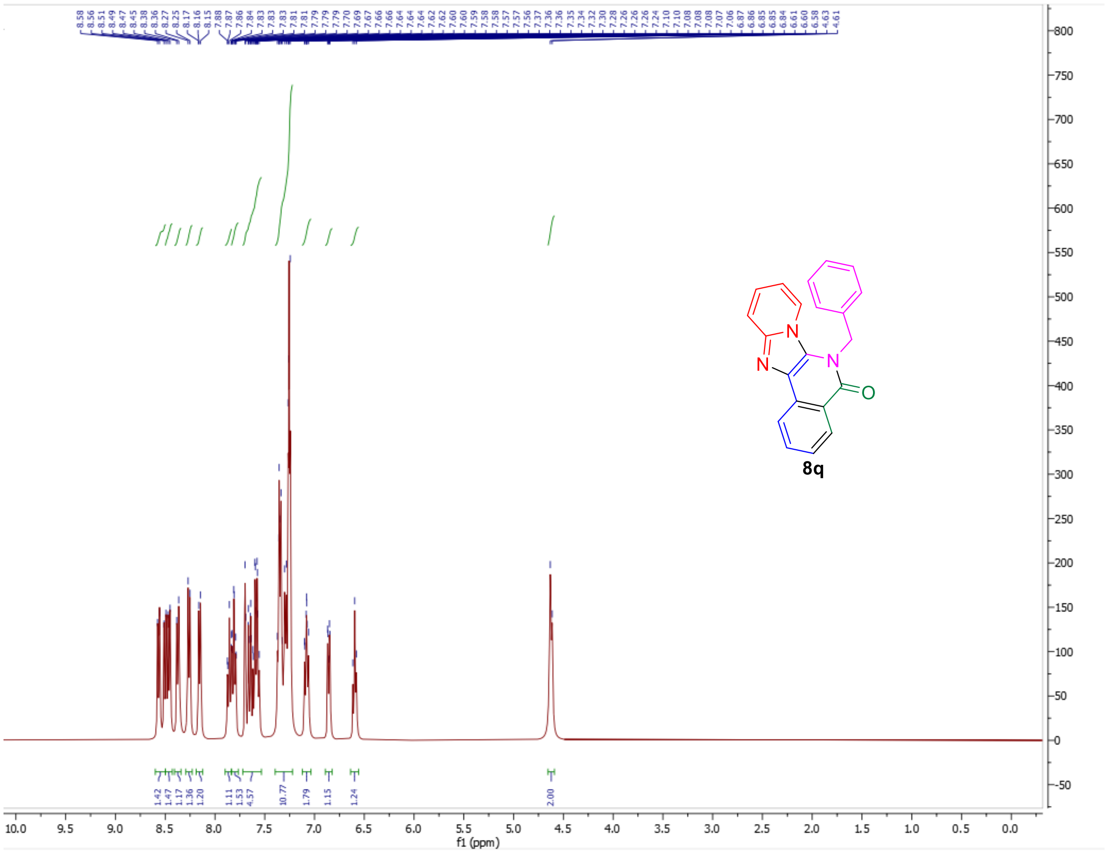

^{1}H NMR of **8q**

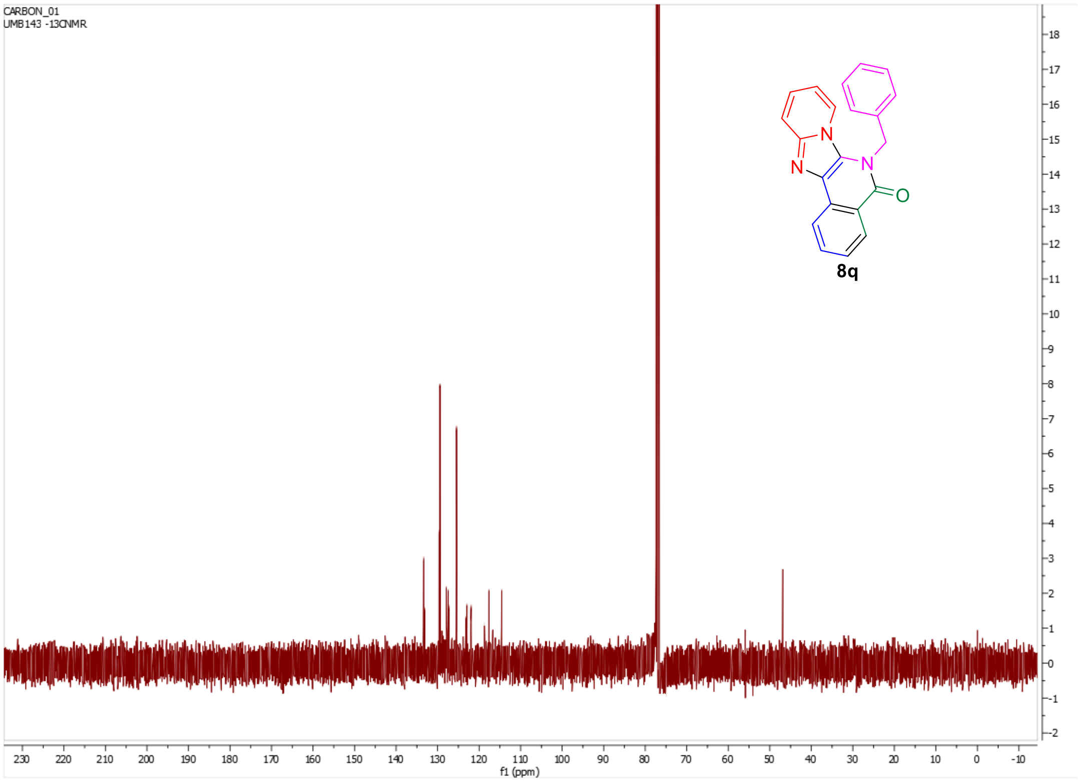

^{13}C NMR of **8q**

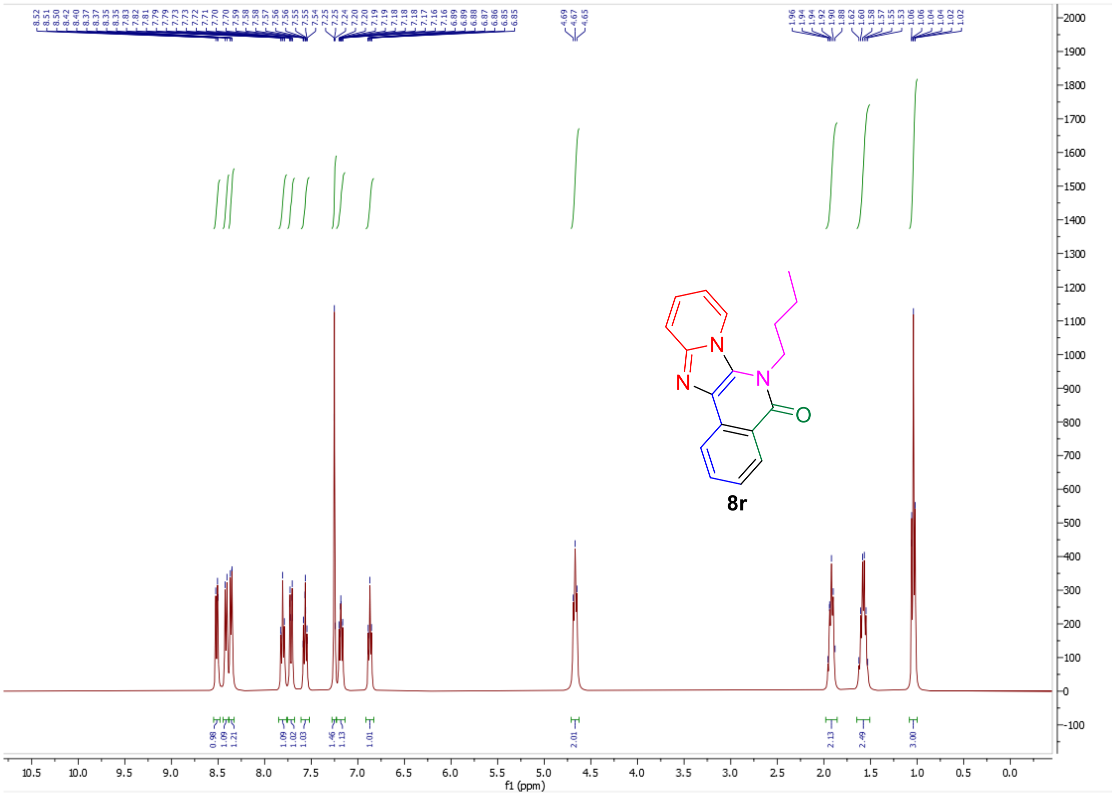

^{1}H NMR of **8r**

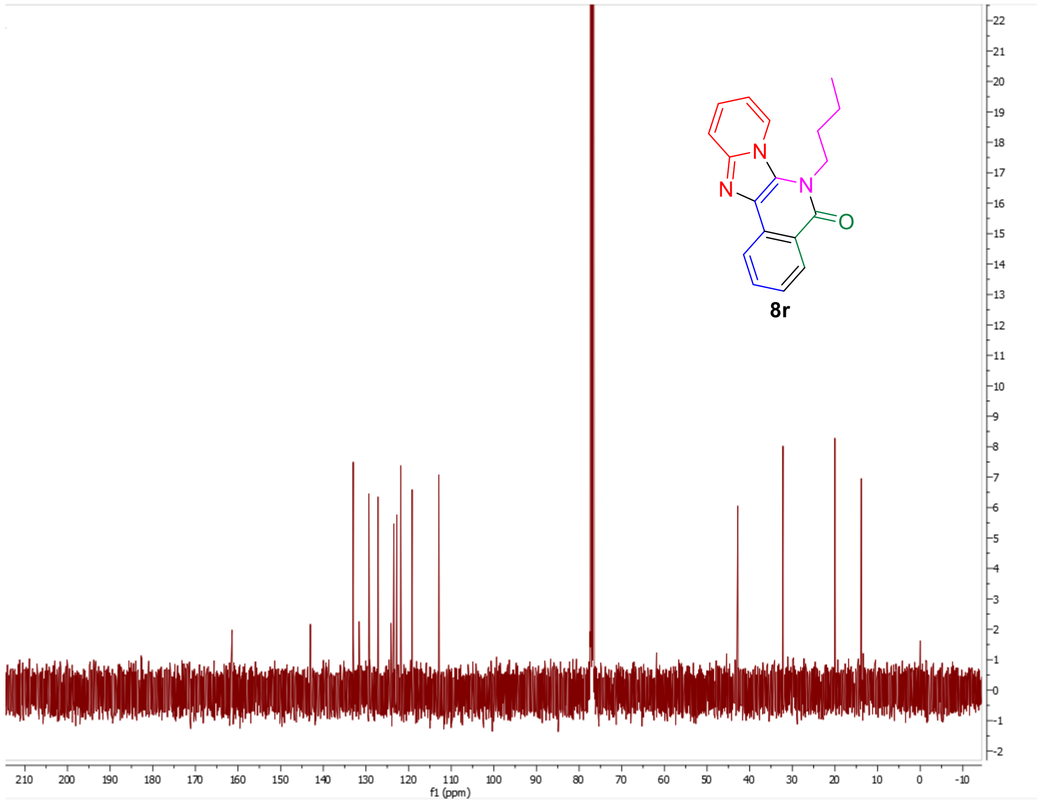

^{13}C NMR of **8r**

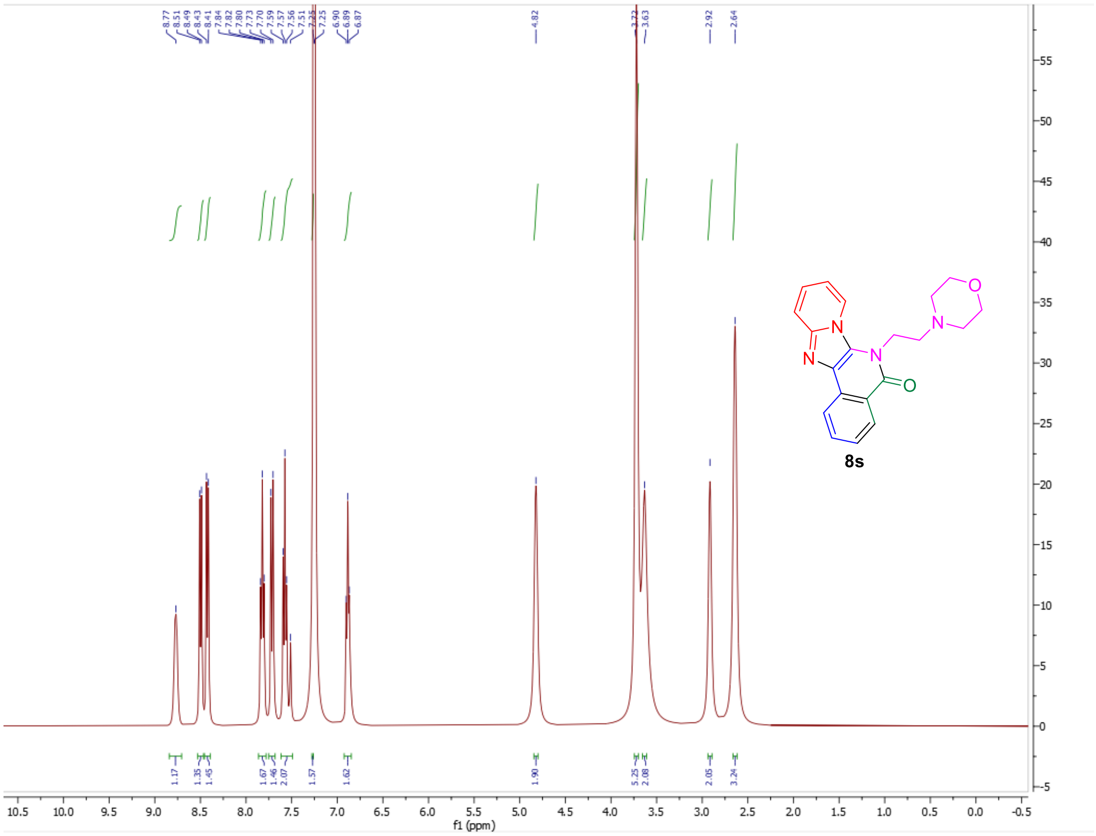

^{1}H NMR of **8s**

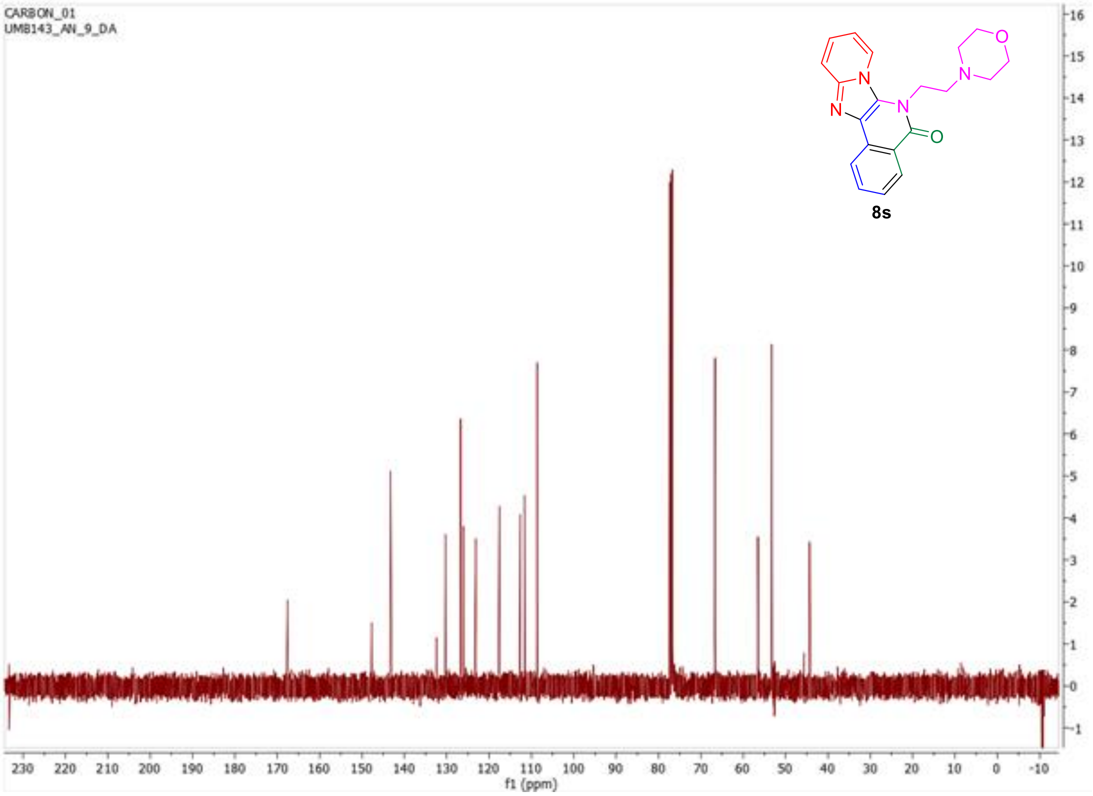

^{13}C NMR of **8s**
